# An Optimized Genotyping Workflow for Identifying Highly SCRaMbLEd Synthetic Yeasts

**DOI:** 10.1021/acssynbio.3c00476

**Published:** 2024-04-10

**Authors:** Timon A. Lindeboom, María del Carmen Sanchez Olmos, Karina Schulz, Cedric K. Brinkmann, Adán A. Ramírez Rojas, Lena Hochrein, Daniel Schindler

**Affiliations:** † Max Planck Institute for Terrestrial Microbiology, Karl-von-Frisch-Str. 10, 35043 Marburg, Germany; ‡ Department of Molecular Biology, University of Potsdam, Karl-Liebknecht-Str. 24/25, 14476 Potsdam, Germany; § Center for Synthetic Microbiology, Philipps-University Marburg, Karl-von-Frisch-Str. 14, 35032Marburg, Germany

**Keywords:** synthetic yeast, SCRaMbLE, synthetic biology, optogenetics, genome rearrangements, recombinases, PCRTags, qPCR genotyping

## Abstract

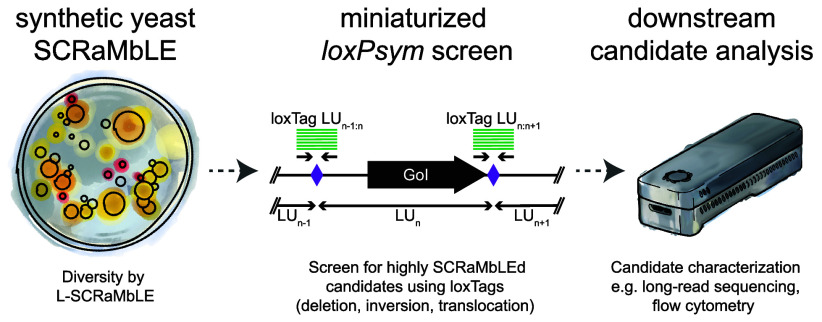

Synthetic Sc2.0 yeast strains contain hundreds to thousands of *loxPsym* recombination sites that allow restructuring of the *Saccharomyces cerevisiae* genome by SCRaMbLE. Thus, a highly diverse yeast population can arise from a single genotype. The selection of genetically diverse candidates with rearranged synthetic chromosomes for downstream analysis requires an efficient and straightforward workflow. Here we present loxTags, a set of qPCR primers for genotyping across *loxPsym* sites to detect not only deletions but also inversions and translocations after SCRaMbLE. To cope with the large number of amplicons, we generated qTagGer, a qPCR genotyping primer prediction tool. Using loxTag-based genotyping and long-read sequencing, we show that light-inducible Cre recombinase L-SCRaMbLE can efficiently generate diverse recombination events when applied to Sc2.0 strains containing a linear or a circular version of synthetic chromosome III.

## Introduction

**Figure 1:**
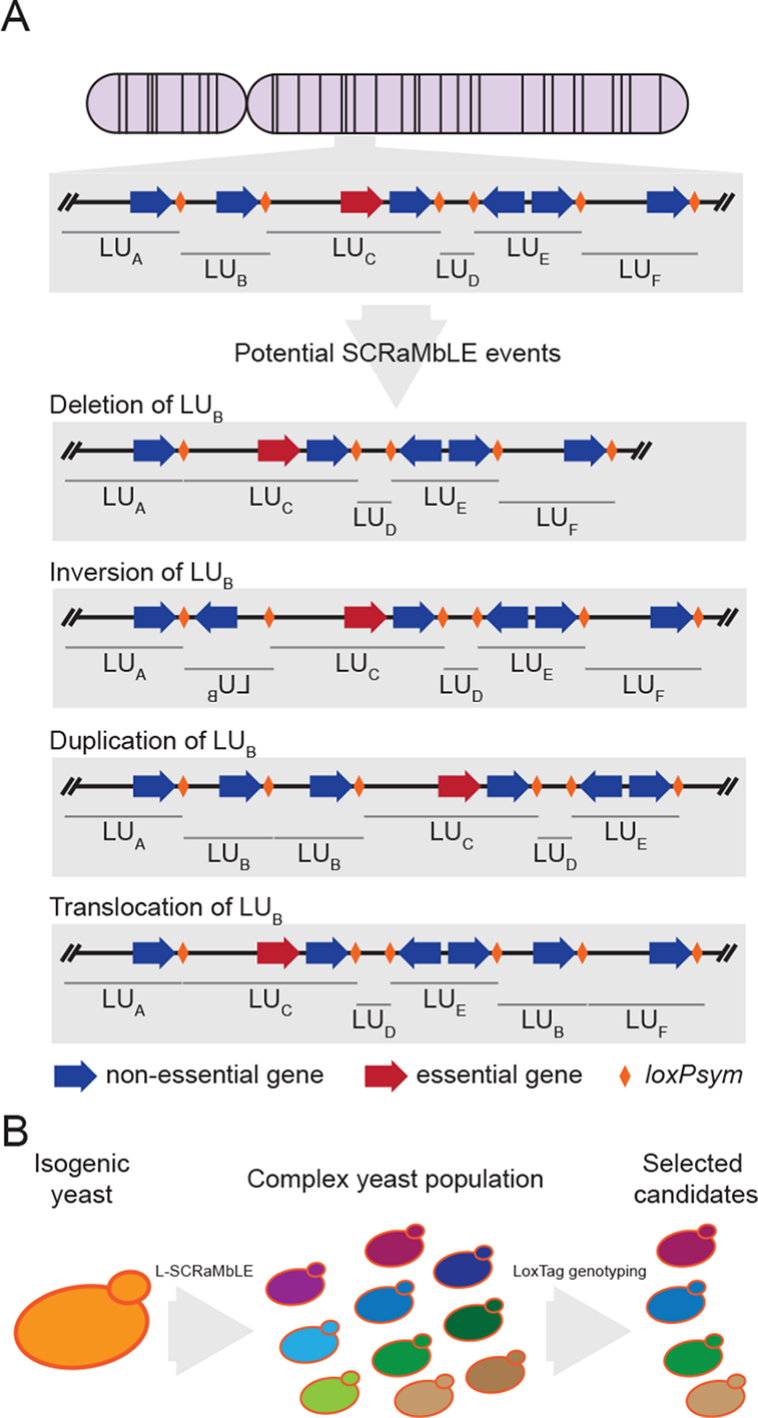
Basics of SCRaMbLE. (A) SCRaMbLE causes deletions, inversions, duplications, and translocations of *loxPsym*-flanked sequences (LUs) in synthetic yeast strains upon Cre activity. Outcomes of the different SCRaMbLE events are shown for LU_B_. (B) SCRaMbLE can turn a single isogenic synthetic yeast strain into a highly diverse population with different properties and characteristics. Subsequently, the complexity of the population needs to be reduced to select relevant candidates for downstream analysis.

Biology is transitioning from a science of observation to a science of engineering. A milestone to achieve this is based on the ability to sequence genomes with great ease and within rapid timeframes.^1,2^ Still, sequence-based knowledge still does not allow one to create life from scratch.^3^ However, the constant progress in improving DNA synthesis has allowed some remarkable studies in rewriting genomes.^4^ The synthetic yeast genome project (Sc2.0) is nearing its completion and is on track to create the first fully synthetic designer eukaryote.^5^ Each of the chromosomes is constructed in a separate strain and subsequently consolidated in one strain to form the final *Saccharomyces cerevisiae* 2.0.^6,7^ Many sequence alterations were incorporated in the design of the Sc2.0 genome, one of the most remarkable might be the implementation of the *Synthetic Chromosome Recombination and Modification by LoxP-mediated Evolution* (SCRaMbLE) system.^6,8^ SCRaMbLE harnesses the ability of site-specific recombinases, in particular, Cre recombinase. Symmetrical *loxP* sites, hereafter referred to as *loxPsym*, were inserted 3 bp downstream of most nonessential genes as well as at other major landmarks (e.g., centromeres). Upon induction of Cre recombinase, Cre can recombine two *loxPsym* and thereby cause stochastic deletions, inversions, translocations, and duplications of *loxPsym*-flanked DNA sequences, so-called loxP units (LU) (Figure 1A).^9^ Thus, a single isogenic strain can be turned into a highly diverse population that can be screened for promising candidates with improved properties by, e.g., whole genome sequencing or metabolic profiling.^10−13^ However, when generating minimal genomes or optimizing bioproduction, preselection of promising candidates based on selection pressure or different phenotypes to reduce the number of candidates for cost- and time-consuming downstream analysis is usually not possible.^9^

To enable an efficient and economical initial analysis for the identification of diverse and highly SCRaMbLEd yeast isolates, thereby reducing the number of candidates for downstream characterization (e.g., whole genome sequencing), we present loxTags (Figure 1B). LoxTags are end-point qPCR primers for genotyping that span *loxPsym* sites to indicate deletions, inversions, and translocations after SCRaMbLE. The genotyping primers for all synthetic Sc2.0 chromosomes were generated using the qTagGer software package, developed by us. LoxTags were used to identify interesting SCRaMbLE candidates from yeast strains containing a circular or linear synthetic version of chromosome III (synIII). In this study, we used the red light-inducible Cre recombinase L-SCRaMbLE on synthetic Sc2.0 strains for the first time. L-SCRaMbLE is built from an optical dimerizer from *Arabidopsis thaliana* fused to a split version of Cre recombinase.^14^ While nonactive in darkness or far-red light conditions, L-SCRaMbLE can be rapidly activated by short red light pulses. Importantly, the Cre activity can be completely switched off again by illumination with far-red light. Our results indicate that 1 h of L-SCRaMbLE activity is sufficient to create a highly diverse population from a single genotype with all possible SCRaMbLE outcomes. Using qPCR screening with loxTags, we could efficiently reduce the number of candidates for long-read whole genome sequencing, which was subsequently used to determine the sequence of the SCRaMbLEd synthetic chromosomes. When analyzing the genomes and performing subsequent validations, we observed genotype alterations that are not directly caused by Cre recombinase such as aneuploidies and whole genome duplications. Based on our results, we propose a standardized analysis pipeline for SCRaMbLEd yeast isolates to prevent unexpected results.

## Results and Discussion

### LoxTag-Based Genotyping for the Identification of Highly Rearranged Yeasts after SCRaMbLE

**Figure 2:**
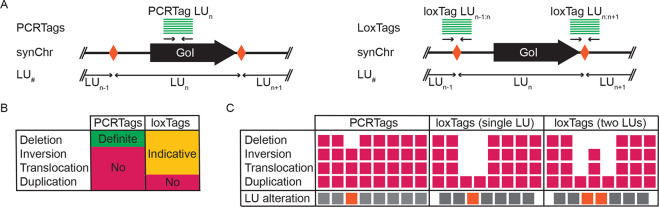
Principles, characteristics, and application of PCRTags and loxTags. (A) PCRTag and loxTag basic principle. PCRTags are encoded sequences within a gene to distinguish synthetic from wild-type DNA. LoxTags generate an amplicon spanning distinct *loxPsyms*. (B) Comparison of PCRTag and loxTag outputs. PCRTags can indicate only the presence or absence of a tested DNA sequence. In contrast to the PCRTags, loxTags can indicate a wider variety of SCRaMbLE events, but the results are only indicative and would need verification, for example, by long-read sequencing. (C) Theoretical results of PCRTag and loxTag analyses of reference sequences with LU alterations. Red boxes indicate positive PCR amplicons. Empty boxes indicate that no PCR amplicon was obtained. Orange boxes indicate true LU alteration. PCRTags detect only deletions of LUs, while loxTags can indicate deletions, inversions, and translocations.

During the construction of the synthetic chromosomes of the Sc2.0 project, PCRTags were designed and integrated into the sequence to distinguish between synthetic and wild-type DNA.^6,8^ PCRTags are short recoded nucleotide sequences within open reading frames (ORF) of genes that allow the generation of specific amplicons either by PCR or in a high-throughput end-point qPCR to prove the presence of synthetic DNA (Figure 2A).^8,15^ Additionally, PCRTag analysis has proven useful for genotype screening of SCRaMbLEd candidates to explain phenotypes as well as to identify promising strains for whole genome sequencing and successive genotype reconstruction.^9,16^ However, current PCRTags provide information only on the presence or absence of a specific amplicon corresponding to a target gene. Thus, they only indicate deletions and do not detect other possible SCRaMbLE outcomes such as inversions, translocations, or duplications. In addition, not every LU contains a PCRTag, which means that some recombination events may not be detected by PCRTag analysis. We therefore designed and tested loxTags for synthetic chromosome III (synIII). LoxTags are genotyping primers that span *loxPsym* sites and allow us to detect most LUs whose order or orientation is altered (Figure 2A). The designed loxTags encompass all LUs except seven which are too small (52 to 191 bp) to generate accurate loxTag primer pairs. In contrast, PCRTags for synIII do not cover 31 LUs. Furthermore, loxTags are diagnostic primer pairs that can give information in regard to potential inversions, deletions, and translocations but do not allow the detection of duplications (Figure 2B). Thus, single LU inversions, deletions, or translocations would be indicated by at least two consecutive missing amplicons, and larger structural variations may have a different pattern of multiple loxTags missing (deletion) or only two distant loxTags missing (large inversions or translocations) (Figure 2C).

To enable a high-throughput design of loxTags, we developed the computer-assisted tool, qTagGer. qTagGer utilizes Primer3^17^ to create a set of diagnostic PCR amplicons for a user-defined motif (e.g., *loxPsym*) within a user-defined input sequence (e.g., a synthetic chromosome). To validate the specificity of designed primers, qTagGer checks the designed primer pairs against the provided reference (for details, see Methods and the GitHub documentation https://github.com/RGSchindler/qTagGer). Using qTagGer, we designed loxTags for all synthetic Sc2.0 chromosomes (Supporting Data S1). Remarkably, qTagGer can be used for any other user-defined motif and is not restricted to *loxPsym*. To prove the versatility of our tool, we also generated primers for the detection of recombination events on the tRNA neochromosome which carries *rox* sites recognized by the Dre recombinase, an orthogonal but not cross-acting recombinase system.^18,19^ We provided all loxTags for the Sc2.0 chromosomes and the roxTags for the tRNA neochromosome in the Supporting Information (Supporting Data S1).

As a proof of concept, we validated the loxTags designed for synIII. SynIII is 272 kb in size, approximately 44 kb shorter than the native chromosome, and holds 98 *loxPsym*.^20^ An initial standard PCR with Taq polymerase (OneTaq 2X Master Mix, NEB) and synIII genomic DNA as a template showed that almost all designed loxTag-primer pairs show specific binding to genomic DNA (Figure S1). Significant off-target amplicons are only visible for *loxPsym* junctions [29:30] and [43:44]. The results were considered promising to establish a dedicated qPCR-based protocol according to Mitchell *et al.*(15) We used the combination of acoustic dispensation and nanoliter bulk dispenser to scale down the individual reactions to 1 μL total volume allowing to screen four isolates within a 384-well qPCR assay (see Methods).^15^ This results in screening costs below 5 € starting from a single colony (including DNA extraction). Importantly, it takes less than 90 min from starting the assay to obtaining the results (excluding the DNA extraction), speeding up analysis and allowing for parallelization.

### Synthetic Chromosomes Show All Possible SCRaMbLE Events after L-SCRaMbLE

**Figure 3:**
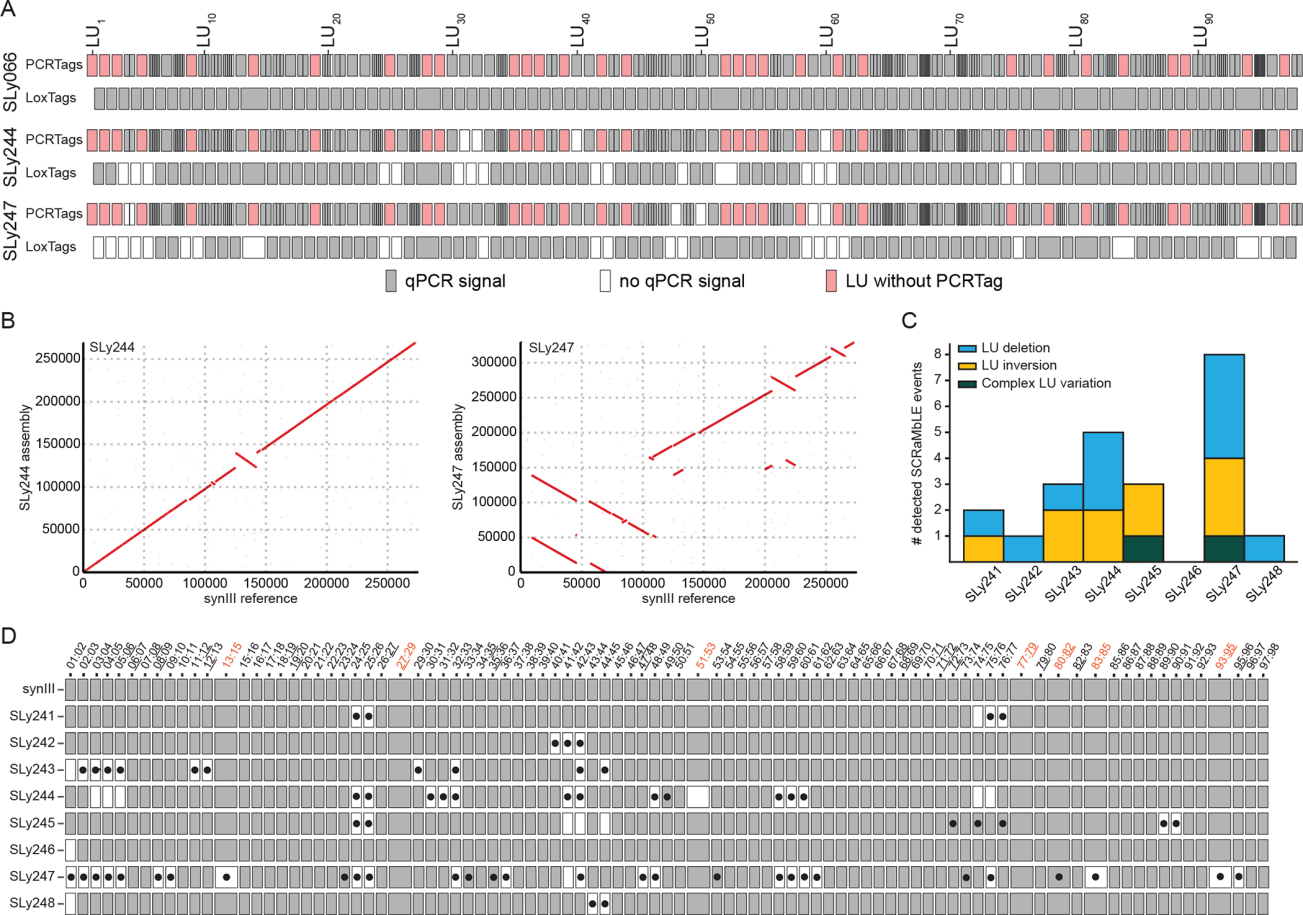
L-SCRaMbLE causes structural variations on chromosome level. (A) Comparison of PCRTags and loxTags by qPCR for the parental strain and two exemplary strains SLy244 and SLy247; full panel for all analyzed strains is provided in the Supporting Information (Figure S4). Gray boxes indicate the presence and white boxes indicate the absence of an amplicon. LUs without PCRTags are indicated pink, and loxTags spanning two LUs are indicated by larger sizes of boxes. (B) Exemplary dot plots for strains SLy244 and SLy247 in comparison with the synIII reference. (C) Overview of the observed SCRaMbLE events for each assembled strain. Deletions and inversions are the most common structural variations in the linear SCRaMbLE derivatives. Complex LU variations are duplications, translocations, and their combination. (D) Comparison of *in silico* end-point qPCR of de novo assembled synIII isolates with the obtained results from the qPCR. White boxes show the absence of loxTag in the qPCR, and black dots indicate absence in the *in silico* data. The *in vitro* and the *in silico* data match in most instances and indicate that loxTag screening is suitable for the selection of SCRaMbLEd isolates.

In order to prove that loxTags can reliably detect diverse SCRaMbLE events, synIII strain SLy066 was SCRaMbLEd for 1 h and subsequently analyzed by qPCR using loxTags. The synIII strain contains the ura3Δ0 allele and a nonfunctional *URA3* at the HO locus.^21,22^ To reduce the risk of unwanted homologous recombination in downstream applications, both alleles were removed in two consecutive rounds of CRISPR/Cas9 genome editing, resulting in intermediate SLy053 and final strain SLy066. Notably, a *URA3* deletion has been described to cause sensitivity to acetic acid.^23^ The L-SCRaMbLE plasmid (pLH_Scr15) was transformed into SLy066, resulting in strain Y1511. Based on an L-SCRaMbLE activity assay (Figure S2), we induced L-SCRaMbLE for 1 h before plating a dilution series to isolate single colonies. Twelve candidates were randomly chosen and subsequently subjected to loxTag analysis. The results indicate a high number of SCRaMbLE events in 8 out of 12 strains (Figure S3). Afterward, the eight highly diverse strains (subsequently termed SLy241–248) were selected for comparison of loxTags and PCRTags. Candidates were solely selected based on loxTag analysis and without consideration of phenotypic data. LoxTags detect additional potential structural variations besides deletions as intended (Figure 3A, full panel Figure S4). Based on our initial results, loxTags seem to be suitable for identifying and selecting highly rearranged synthetic yeast genomes for subsequent analysis. To precisely determine the occurring SCRaMbLE events, the same strains were submitted to long-read sequencing. Long-read sequencing of SLy241–248 resulted in an average of 42-fold coverage for the linear synIII strains (Table S1). *De novo* assembly using canu^24^ resulted in a single synIII derivative for each strain. To visualize the structural variations, we used dot plots allowing for comparison with the parental strain (Figures 3B and S5). All strains examined show changes compared to the reference in the form of deletions, inversions, duplications, and translocations, respectively, with the exception of strain SLy246, which shows no alterations (Figures 3C and S5). It was later validated that this initial loxTag screen of SLy246 had an experimental error; notably, this was the only case where the qPCR reaction malfunctioned. Deletions and inversions form the majority, accounting for approximately 90% of the observed events, and are more common in contrast to complex structural variations (duplications, translocations, and their combinations). To compare the results obtained by loxTag screening to the sequencing data, we generated the custom Python script “visLoxP” simulating *in silico* amplicons of our assembled genomes (Figure 3D). The loxTags results for five of the eight strains examined are in good agreement with the sequencing results. However, for strains SLy244, SLy245, and SLy247, differences are observed between the loxTag screening and the *in silico* result of the *de novo* assembled SCRaMbLEd synIII chromosomes. SLy244 shows a high number of false negative loxTags (6 of 14 events). SLy245 shows three false positive and three false negative loxTags, respectively. SLy247 shows six false positive loxTags. Importantly, these three strains are the most complex SCRaMbLEd synIII strains, which could indicate that the *de novo* assembly may not be correct. The general *de novo* assembly problem will be discussed in detail in the next section. Furthermore, we used pulsed-field gel electrophoresis (PFGE) to confirm that the sizes of *in silico* assembled SCRaMbLEd synIII chromosomes correspond to the actual sizes (Figure S6). Importantly, synIII and chrVI have similar sizes of 270.2 kb and 272.9 kb, respectively, resulting in a double band in most cases. Nevertheless, PFGE is consistent with our long-read assembly results. After the initial qPCR methodology was improved (*cf*. Figure S3) that we used to select the candidates for subsequent long-read sequencing, the results of end-point qPCR and *de novo* assembled synIII performed with the optimized qPCR methodology indicate that loxTags are a suitable screening tool and well suited to select diverse candidates for downstream applications (Figure 3D).

### Circular Synthetic Chromosomes Show an Increase in Structural Variations

**Figure 4:**
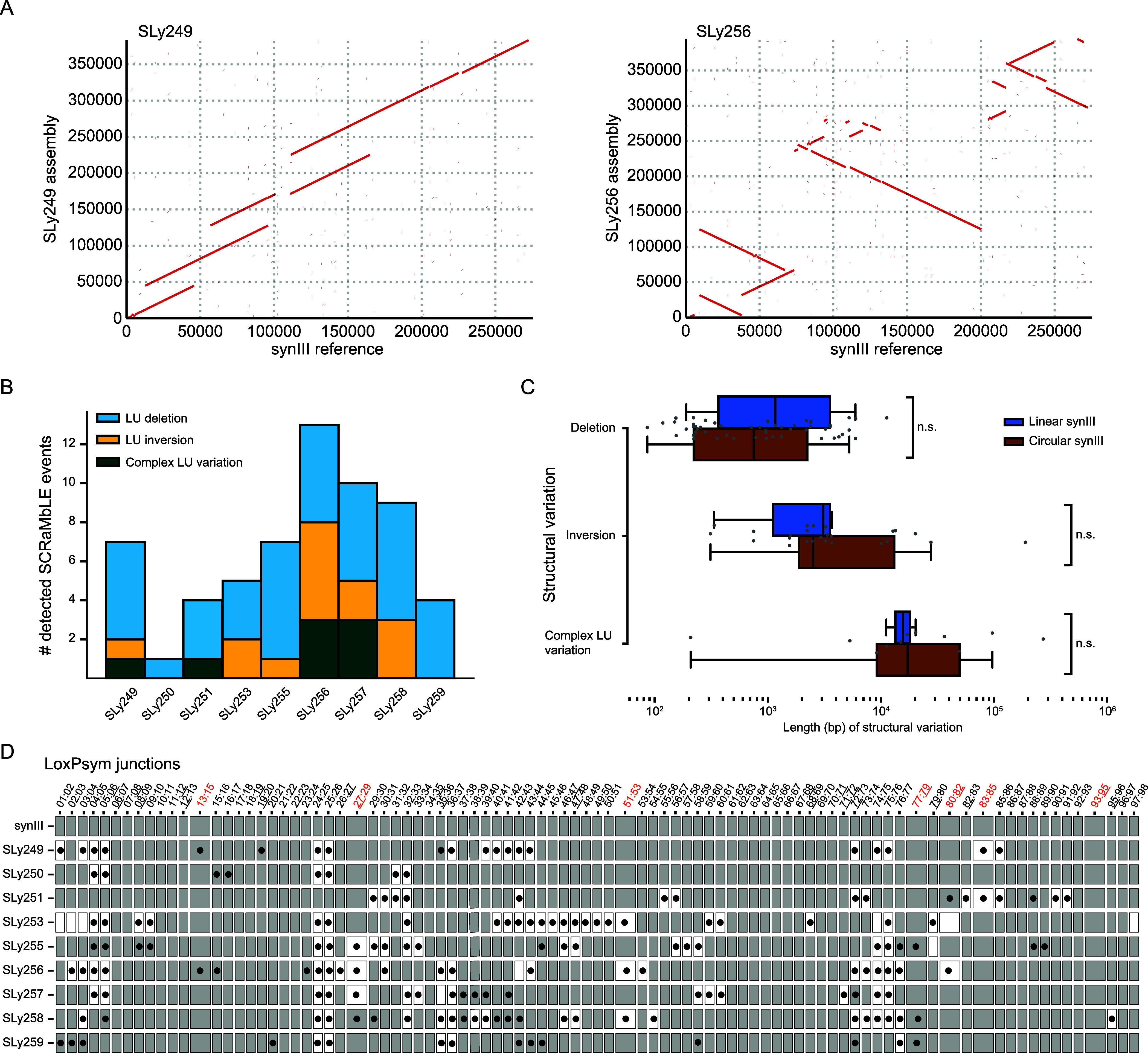
Circular chromosomes show a higher degree of L-SCRaMbLE activity. (A) Exemplary dot plots for SCRaMbLEd circular synIII strains SLy249 and SLy256 in comparison to the synIII reference SLy117. All types of SCRaMbLE events caused by L-SCRaMbLE are observed. Dot plots for all circular synIII strains are visualized in Figure S7. (B) Overview of the observed SCRaMbLE events for each assembled strain. Complex LU variations are duplications, translocations, and their combination. (C) Comparison of lengths of structural variations observed after SCRaMbLE in linear and circular synIII isolates. No significant (ns) differences (Mann–Whitney U test) can be observed in this data set. (D) Comparison of *in silico* end-point qPCR of assembled synIII derivatives with the obtained results from the qPCR using visLoxP. White boxes show the absence of loxTag in the qPCR, and black dots indicate absence in the *in silico* end-point qPCR.

Previous studies showed that circular synthetic chromosomes exhibit an increased number of SCRaMbLE events.^25^ Therefore, we circularized the linear synIII of strain SLy066 using CRISPR/Cas9-guided homology-directed repair by providing a repair template and two gRNAs cutting in the subtelomeric repeats of the left and right arms, respectively, resulting in strain SLy117. The circular synIII created within this study is reduced in size by approximately 500 bp in comparison to synIII but holds the same number of 98 *loxPsym* sites and shows no obvious phenotypic changes compared to the linear synIII. The circularity of synIII was verified using PCR, long-read sequencing, and PFGE (*cf*. Figure S6). The L-SCRaMbLE plasmid (pLH_Scr15) was transformed into SLy117, resulting in strain Y1512. Y1512 was induced via red light using the same settings as for Y1511, and cultures were plated after SCRaMbLE. Twenty-four single colonies were randomly chosen and subjected to loxTag analysis (Figure S7). From these, 11 candidates (strains SLy249–259) were selected and subjected to long-read sequencing. The genome coverage for the circular synIII strains was on average 29-fold (Table S1). While all tests performed for the analysis of the linear synIII derivatives support the correctness of our synIII assemblies, the circular chromosomes show highly complex structural variation, which may not be solved properly with the available long-read *de novo* assemblers. While canu^24^ was sufficient to assemble the linear chromosomes, it was previously reported to have poor performance when assembling circular chromosomes.^26^ Our attempts using canu to assemble the circular synIII strains were not successful. Therefore, we used Flye, which, in the case of the circular synIII derivatives, provided the most likely solutions for the SCRaMbLEd synIII chromosomes.^27^ Flye has previously been shown to perform well for circular chromosomes.^26^ The strains SLy252 and SLy254 show highly complex rearranged synIII chromosomes, and all our assembly attempts for these strains are most likely incorrect, despite the general coverage for the samples being >15-fold (Table S1), which should be sufficient to perform whole genome assembly with long-read sequencing data. For this reason, we exclude our synIII assemblies of SLy252 and SLy254. However, in line with previous reports, we observed a lower abundance in whole genome sequencing experiments on circular chromosomes, which may limit our assembly attempts.^28^ Our data highlight that dedicated software tools are necessary to decode complex SCRaMbLEd synthetic chromosomes. Nonetheless, based on the results obtained, we can confirm that circular synthetic chromosomes show a higher degree of structural rearrangement compared to the linear chromosomes, which is depicted in the corresponding dot plots and structural variation histogram (Figures 4A,B and S8).^25^ Compared with the quantification of SCRaMbLE events observed in linear strains, the analyzed candidates of the circular strains show a significantly higher number of recombination events (*cf*. Figures 3B and 4B). For the circular synIII strains, the majority of approximately 86% are deletions (63%) and inversions (23%). In our experimental setup, the average size for Cre-mediated deletions, inversions, and duplications is 1.4, 19.4, and 55.6 kb, respectively. We did not observe significant differences in the length of SCRaMbLE variations between the linear and circular strains (Figure 4C). We admit the number of analyzed strains is relatively low, and a systematic study would be necessary to investigate potential differences in detail. To conclude if the loxTag-based selection of the circular synIII candidates was reasonable, we used again visLoxP to compare our loxTag analysis with the obtained synIII assemblies (Figure 4D). The qPCR and *in silico* data are in good agreement for the SCRaMbLEd yeast strains with generally higher confidence *de novo* assemblies (Figure S9; SLy249 to SLy251, SLy253, and SLy256). The strains SLy255 and SLy257–SLy259 show the highest differences (mostly false negatives) when comparing loxTag screening and the resulting *de novo* assembly. The discrepancies are most likely because of incorrect *de novo* assemblies caused by low N50 values (the N50 value is the median of contig length containing 50% of the whole genome assembly) and the generally lower read abundances of circular chromosomes (details in Figure S9). This highlights the need for improved *de novo* assembly tools for highly SCRaMbLEd synthetic yeast strains, the major problem most likely being highly repetitive regions and long tandem repeats. However, for the strains with higher confidence *de novo* assemblies (Figure S9; SLy249 to SLy251, SLy253, and SLy256), the results of loxTag screening and *in silico* prediction of assemblies are in good agreement. We conclude that loxTags are a useful screening tool for the selection of highly SCRaMbLEd synthetic yeast candidates. This is particularly important when limited sequencing capacity is available to ensure that highly SCRaMbLEd strains are selected and, in addition, that highly similar strains are avoided.

Interestingly, despite our sample size, we observed that certain LUs of the linear and circular SCRaMbLEd synIII strains showed increased recombination activity (Figures S3 and S7). For example, LU25 corresponds to the region downstream of *KCC4* and upstream of *NSF1*. It is 186 bp long and has no annotated function. Presumably, it can get easily rearranged (e.g., deleted or inverted) based on the close proximity or the accessibility of the *loxPsym* sites. In a recent study, short-read deep sequencing was performed on SCRaMbLEd populations with 5.5 synthetic chromosomes. The short-read sequencing data were investigated in regard to changes of sequence flanking *loxPsym*. The authors could show that recombination frequency correlates with *loxPsym* accessibility based on ATAC-seq data.^31^ Combining deep sequencing data with information about individual strains may in the future provide details on the likelihood of SCRaMbLE events. These data could potentially be used to approximate how large a SCRaMbLEd population needs to be to obtain candidates with desired or rare characteristics.

### SCRaMbLE Can Have a Global Impact on Yeast Karyotypes

**Figure 5:**
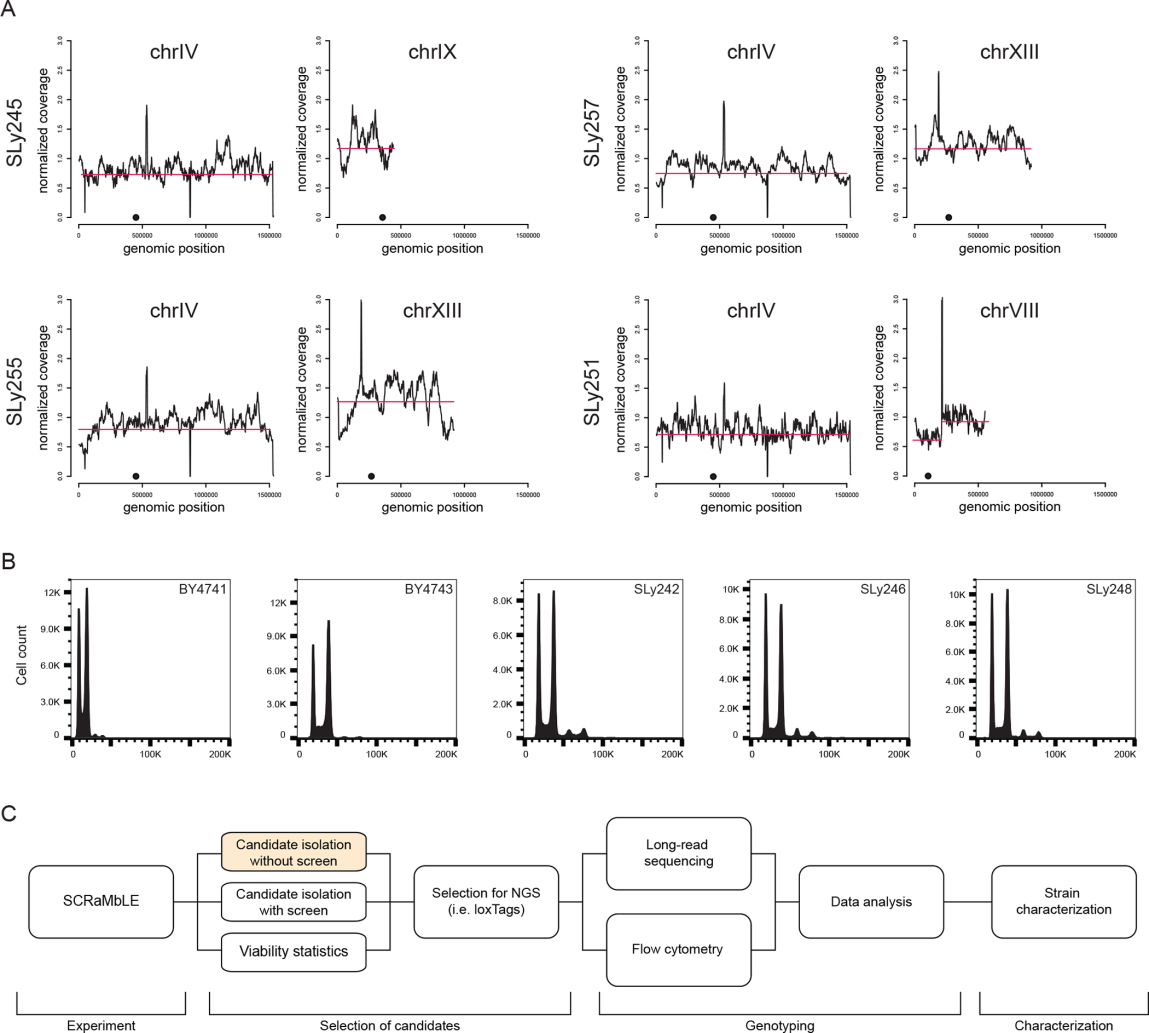
Complex karyotype alterations as an unintended result of SCRaMbLE. (A) Aneuploidies and segmental chromosome amplification indicated by normalized coverage of Nanopore sequencing reads. Red line visualizes coverage shift, and black dot indicates the position of centromere. Whole genome coverage plots are provided in the Supporting Information (Figure S10). (B) Flow cytometry identifies SLy242, SLy246, and SLy248 to have the same DNA content as the diploid wild-type strain BY4743, indicating a whole genome duplication. Whole genome coverage plots are provided in the Supporting Information (Figure S10), showing that whole genome sequencing does not detect whole genome duplications. (C) Best practice workflow to perform and analyze SCRaMbLEd synthetic yeast strains. In the cases where screening for an improved phenotype is not possible (highlighted orange), alternative methods such as loxTag genotyping presented here are necessary to reduce the number of candidates and ensure SCRaMbLEd isolates are identified for the downstream analysis.

When we analyzed the sequencing data beyond the assembly of synIII, we found an increased copy number for individual chromosomes in three instances. The abundance of reads for these chromosomes approximately doubles in comparison to the other chromosomes, indicating +1 aneuploidies (Figures 5A and S10). SLy245, SLy255, and SLy257 contain aneuploidies of chromosomes IX, XIII, and XIII respectively. Further, long-read sequencing results of SLy251 show a segmental duplication of roughly half of chromosome VIII. Coverage plots for all chromosomes for the parental strain and the isolated strains with observed alterations are provided in the Supporting Information (Figure S10). Based on our observation and previous reports about whole genome duplication as a common stress response in yeast,^29^ we decided to investigate the ploidy of all strains. Therefore, we performed flow cytometry of exponentially growing cells of strains SLy241–259 in comparison to known haploid and diploid reference strains (BY4741 and BY4743) (Figures 5B and S11). By doing so, we discovered whole genome duplication for isolates SLy242, SLy246, and SLy248 carrying linear synIII chromosomes. Remarkably, a whole genome duplication is not visible in whole genome sequencing data (Figure S10F–H). All circular synIII derivatives contain haploid genomes. Importantly, we have not observed any integration or recombination between synIII and the native chromosomes, proving the targeted action of Cre recombinase. However, our results and other studies show that SCRaMbLE can have a variety of impacts on yeast karyotype, ranging from segmental to whole chromosome and genome duplications.^25,28^ It was previously reported that aneuploidies and whole genome duplication may have an impact on yeast growth in the presence of hydroxyurea.^19,28^ Therefore, we performed growth assays on solid synthetic complete (SC) medium for the selected strains in comparison to the parental strain to test whether this may serve as a simple test to detect aneuploidies in contrast to flow cytometry. However, it was observed that the strains with aneuploidies and whole genome duplication showed reduced growth fitness on hydroxyurea-containing plates (Figure S12), leading to the conclusion that this simple phenotyping assay, in contrast to flow cytometry, is not useful for the intended purpose (Figures S11 and S12).

Based on our results, we suggest a standard workflow to characterize SCRaMbLEd yeast isolates (Figure 5C). The workflow starts with the SCRaMbLE experiment, including viability statistics to check for Cre activity and the subsequent selection of isolates for detailed analysis. If selection for an improved phenotype is possible (e.g., improved growth on stress medium), the number of candidates can be reduced at this stage. However, it is important to compare the selected condition with an appropriate control condition to validate the enrichment. In the case of the absence of selection pressure, phenotypes, or large numbers of isolates, loxTags help to identify relevant candidates. Reducing the number of candidates streamlines the subsequent long-read sequencing experiments and analysis. Notably, long-read sequencing seems to be necessary to assemble complex rearranged synthetic chromosomes and may not solve all analyzed isolates. Subsequently, sequencing data should be screened for genome alterations such as +1 aneuploidies or segmental duplications. In parallel DNA content measurements with flow cytometry can identify whole genome duplication, which cannot be detected by whole genome sequencing data. PFGE can validate the karyotype and can be supportive in the case of linear chromosomes to validate the assembly size. Strains characterized in such a manner can afterward be further investigated in regard to their biology. A standardized workflow becomes particularly relevant if observed genotype alterations are planned to be transferred to biotechnologically relevant producer strains or other yeast species.

## Conclusions and Perspectives

Here, we showed for the first time that the red-light-regulated Cre recombinase L-SCRaMbLE is capable of causing all types of structural variations in Sc2.0 strains holding a linear or circular version of synIII. By analyzing only a small number of SCRaMbLEd strains, we were able to identify several candidates exhibiting multiple and diverse recombination events. Remarkably, candidates were chosen without any selection for Cre activity, e.g., the ReSCuES system, which harnesses an auxotrophic marker switch to select for strains where Cre has been active.^30^ This proves that L-SCRaMbLE is an excellent tool to tightly regulate Cre activity not only at the plasmid level but also at the genome level. Furthermore, we provide evidence that an induction of 1 h is sufficient to create a highly diverse yeast population.

To allow identification of promising SCRaMbLE candidates for subsequent characterization without selection pressure or phenotypic analysis, we present an efficient high-throughput analysis workflow. To enable qPCR-based screening of SCRaMbLEd yeast isolates in order to identify diverse highly recombined strains, we established loxTag genotyping primers which can detect deletions, inversions, and translocations. LoxTag primer pairs were generated for all synthetic yeast chromosomes based on our computational tool qTagGer (Supporting Data S1). When tested on a linear and circular version of synIII, we could show that our loxTags detect a broader range of SCRaMbLE events compared to the Sc2.0 PCRTags that only indicate deletions. Thus, loxTags provide an improved decision-making tool to identify suitable candidates for subsequent characterization by reducing the number of identical candidates and identifying diverse SCRaMbLEd candidates for the subsequent characterization steps. LoxTags work economically and time efficiently, in particular, if the reaction is downscaled by laboratory automation. With our approach, we can screen four synIII candidates on a single 384-well qPCR run in 1 μL reactions, lowering the costs to less than 5 € per strain (including DNA extraction, etc.) and obtaining results within 90 min (excluding DNA extraction). It is worth mentioning that a screening method selecting for the absence of a PCR amplicon is not ideal, but on this global scale, it is the only strategy to reduce the set of candidates for long-read sequencing, a costly and time-consuming process in comparison to qPCR. Therefore, we are sure that loxTags will be a helpful resource for the community. Moreover, the newly established tool qTagGer, which generates genotyping primers, may be broadly applied in other aspects where rearrangements at specific nucleotide sequences need to be studied.

## Methods

### Strains and Growth Conditions Used in This Study

All yeast strains were derivatives of S288c and grown at 30 °C unless otherwise specified. A full list of yeast strains is provided in Table S2. The standard *Escherichia coli* TOP10 strain (Invitrogen) was used for propagation and archiving of plasmid DNA. Yeast cells were grown in either YEP/YPD media (10 g/L yeast extract, 20 g/L peptone, and with or without 0.64 g/L tryptophan) or SC media (1.7 g/L yeast nitrogen base without amino acids and without ammonium sulfate, 5 g/L ammonium sulfate, 1.47 g/L SC triple drop-out -His, -Leu, -Ura; all components used are from Formedium) lacking the indicated amino acids, both supplemented with 2% glucose if not stated otherwise. Standard solid media contained 2% agar and cultures were incubated at 30 °C. Bacterial cultures were grown in LB medium at 37 °C with 100 μg/mL ampicillin. Bacterial and yeast liquid cultures were cultivated according to standard procedures in glass tubes on a custom-built roller drum (similar to New Brunswick TC-7) or on shaking incubators (Innova 42R, New Brunswick) at 200 rpm.

### Plasmids and Oligonucleotides Used in This Study

All plasmids used in this study are listed in Table S3, and the plasmid files of the created plasmids are provided as Genbank files (Supporting Data S2). Oligonucleotides were ordered and synthesized by Integrated DNA Technologies in 25 or 100 nM scale as standard desalted oligonucleotides (Table S4). PCRTag primers were synthesized according to Annaluru et al.^20^ LoxTags are listed separately in the Supporting Information; Table S5 lists the physical synIII loxTags, and Supporting Data S1 contains all predicted sequences (incl. synIII).

### DNA Purification and Plasmid Extraction

Plasmid DNA and PCR product purification was performed as previously according to open-source magnetic bead purification procedures.^32,33^ Yeast DNA isolation was performed using a modified cetyltrimethyl ammonium bromide extraction method applying glass beads^34^ and a FastPrep96 bead beater for cell rupture. Briefly, cells were cultivated overnight in respective media, 1 mL of culture was harvested at 13,400 × *g* for 1 min in 2 mL screw cap tubes and resuspended in 1 mL of extraction buffer (2% CTAB, 100 mM Tris, 1.4 M NaCl and 10 mM EDTA, pH 8.0), and 425–600 mm glass beads were added approximately to 0.3 mL scale bar. Subsequently, tubes were processed either by vortexing at the maximum speed for 15 min with the Vortex Genie 2 (Scientific Industries) with a 24 multitube holder adapter or by placing the tubes into a FastPrep96 (MP Biomedicals) with custom-made adapters to process up to 2 × 48 tubes simultaneously. Cell rupture was performed with the following settings: 1400 rpm for 15 min. Screw cap tubes were used to omit potential cross-contamination caused by cell rupture in 96-well deep-well plates considering the sensitivity of qPCR assays. Tubes were then incubated for 10 min at 65 °C. Vortexing and incubation were then repeated once in order to improve yield. After the second incubation, tubes were centrifuged at 13,400 × *g* for 1 min. The supernatant was transferred to 2 mL tubes containing 16 μL of 25 mg/mL RNaseA and incubated at 37 °C for 15 min. Afterward, 700 μL of chloroform was added, and the tube was mixed by inversion and centrifuged at 13,400 × *g* for 2 min. The aqueous phase (∼700 μL) was transferred to a 1.5 mL reaction tube and 0.6 volumes of isopropanol was added. The tube was mixed by inversion and centrifuged as before. Isopropanol was discarded, and the pellet was washed in 500 μL of 70% ethanol and centrifuged for 2 min at 13,400 × *g*. The supernatant was discarded, the tubes pulse-centrifuged and residual alcohol was removed using pipet tips, and the pellet was dried with an open lid for 2–5 min at room temperature. The pellet was resuspended in 50 μL of dH_2_O and stored at −20 °C until further use.

### Yeast Transformation

Yeast strains were transformed using a modified protocol described by Gietz and Woods.^35^ Briefly, yeast strains were inoculated into 10 mL of liquid media and incubated with rotation at 30 °C overnight. Overnight cultures were then reinoculated to OD_600_ = 0.1 and incubated with rotation to a target OD_600_ of between 0.5 and 1. Cells were then centrifuged at 2103 × *g* for 5 min, washed with 10 mL of dH_2_O, and centrifuged again. Cell pellets were washed with 10 mL of 0.1 M LiOAc and centrifuged at 2103 × *g* for 5 min. Fifty μL of cells were mixed with 5 to 10 μL of transforming DNA, 36 μL of 1 M LiOAc, 19 μL of dH_2_O, 25 μL of 10 mg/mL herring sperm carrier DNA, and 240 μL of 44% PEG 3350. Transformations were incubated at 30 °C for 30 min before adding 36 μL of DMSO and then subjected to heat shock at 42 °C for 15 min. Cells were pelleted and incubated in 400 μL of 5 mM CaCl_2_ at room temperature for 10 min and then plated onto selective media.

### Construction of gRNA Plasmids and Repair Templates

Preparation of gRNA plasmids was done according to the protocol provided by the Ellis Lab (https://benchling.com/pub/ellis-crispr-tools).^36^ Briefly, the generation of gRNA plasmids was accomplished by insertion of annealed oligos into the destination plasmid pWS082 via Golden Gate cloning. Guide RNAs were designed using the online tool CHOPCHOP.^37^ Repair templates were generated by either using overlap extension PCR or subcloning of multiple fragments into pSL0014 via Golden Gate cloning and subsequent amplification with oligos SLo0101 and SLo0138. Overlap extension PCR was performed as follows: 5′ and 3′ DNA segments flanking the area to be edited were amplified from genomic DNA using the corresponding designed oligonucleotides (Table S4). The resulting fragments contain an overlap with matching *T*_m_ for the 5′ amplicon forward and 3′ amplicon reverse primer to fuse both fragments in a subsequent PCR. One μL of the 5′ and 3′ amplicon was used in the second PCR as a template without purification, resulting in the repair template amplified by the respective 5′ forward and 3′ reverse primers. The repair templates for CRISPR/Cas9 contained at least 300 bp for homologous recombination in yeast. Prior to yeast transformation, the gRNA plasmid(s) were linearized for 1 h by digestion with EcoRV, and repair templates were generated using PCR.

### Construction of synIII Strain Derivatives

SynIII contains the *ura3*Δ*0* allele and a remaining *URA3* marker at the HO locus.^21,22^ Both *URA3* alleles were removed using CRISPR/Cas9 in conjunction with one or three gRNAs targeting the *ura3*Δ*0* allele (pSL0370) and *URA3* (pSL0096–98), respectively, and the supplied donor DNA serving as the repair template resulting in strains SLy053 and subsequently SLy066. SLy066 was subsequently used for synIII ring chromosome formation using CRISPR/Cas9 in conjunction with two gRNAs (pSL0271–272) targeting the subtelomeric repeats and a donor DNA serving as a repair template (PCR amplified from pSL0270) resulting in strain SLy117 with a circular synIII. CRISPR/Cas9 editing in yeast was performed following the protocol outlined at https://benchling.com/pub/ellis-crispr-tools.^36^ Briefly, linear fragments encoding Cas9, gRNA(s), and repair template(s) were cotransformed into yeast strains using the aforementioned yeast transformation method. Plasmids containing gRNAs, DNA sequences used as the repair template, and circular synIII are provided as GenBank files in the supporting data (Supporting Data S2).

### Light-Induced Recombination Experiments

For recombination experiments, yeast strains Y1510–Y1512 were inoculated in 2 mL of SC-Leu in culture tubes and incubated by shaking for 24 h (30 °C, 220 rpm). From this preculture, main cultures were inoculated to an OD_600_ ≈ 0.1 in 500 μL of fresh SC-Leu medium in 24-well plates with a transparent bottom (product no. 303008, Porvair Science Ltd., Norfolk, U.K.) covered with sterile aluminum sealing foil. Induced samples contained 25 μM phycocyanobilin (SC-1800, SiChem GmbH, Bremen, Germany). All samples were inactivated by a 1 min far-red light pulse (740 nm, 69 W/m^2^) and afterward grown for 6 h in darkness (30 °C, 220 rpm). After that, noninduced samples were further maintained in darkness for 1, 2, or 4 h. Induced samples were irradiated with a 5 min red light pulse (660 nm, 28 W/m^2^) and afterward grown for 1, 2, or 4 h with 10 s red light pulses applied every 5 min. Light-induction experiments were conducted in a custom-made Light Plate Apparatus (LPA) device.^38^ LPAs were equipped with 660 nm LEDs (product no. L2-0-R5TH50-1, LEDsupply, Randolph, VT, USA) and 740 nm LEDs (product no. MTE1074N1-R, Marktech Optoelectronics Inc., Latham, NY, USA). All light-sensitive manipulations were done under green safelight.

### Lethality Assays of SCRaMbLEd Yeast Cultures

For lethality assays, cultures were treated as described above in “Light-induced recombination experiments”. Hundred μL of each culture was used to prepare sequential dilutions in a 96-well microtiter plate and 20 μL of each dilution was spotted on SC-Leu plates. Assays were performed in three independent replicates.

### qPCRTag Generation

Diagnostic qPCR primer pairs for synIII were designed by using a prototype of qTagGer and manually curated to remove redundancies. qTagGer later evolved into a command line tool for *S. cerevisiae* written in Python, which could be expanded to other organisms. Briefly, qTagGer integrates Primer3 for primer design^39,40^ with a masking function for *S. cerevisiae*(41) to exclude error-prone regions before primer design is performed. Off-target detection is performed by applying bowtie^42^ to detect all sequences with multiple matches in the genome with a defined mismatch value (default value ≤4 bp). All off-target and Primer3 configuration parameters can be customized in the corresponding .yaml file. qTagGer was subsequently used to predict loxTags and roxTags for all synthetic chromosomes; references used are listed in Table S6. LoxTags and roxTags were manually curated and are provided in the Supporting Information (Supporting Data S1). qTagGer is maintained and available at GitHub (https://github.com/RGSchindler/qTagGer).

### qPCR Tagging and Data Analysis

qPCR analysis of both loxTags and PCRTags was done by adapting an earlier established protocol.^15^ The methodology described here represents the optimized procedure after the initial loxTag screen. LoxTag and PCRTag primer pairs were dispensed using an Echo525 instrument (Labcyte). 25 nL of 50 μM premixed forward and reverse primers was dispensed in 384 PCR plates (Sarstedt, 72.1984.202); 25 nL was not included in the total volume calculation. Primer plates were prepared in bulk and stored for up to 4 weeks. To perform the 1 μL qPCR assay, 1× Luna Universal qPCR Master Mix (M3003, NEB) containing 2 ng/μL template DNA was dispensed using a nanoliter dispenser (Cobra 4-channel dispenser, ARI). Plates were spun down at 500 × *g* for 5 min followed by sealing at 180 °C for 2 s with optical clear permanent seal (Agilent, 24212-001) using the Plateloc Thermal Microplate Sealer (Agilent). qPCRs were run using Applied Biosystems QuantStudio 5. Samples were preincubated at 50 °C (1.6 °C/s) for 2 min followed by 1 min 95 °C (2.57 °C/s). A two-step PCR reaction was performed with 30 cycles each 95 °C (2.57 °C/s) 1 s and 67 °C (2 °C/s) 1 min, with single acquisition. Melting curve was acquired from 97 °C (0.1 °C/s) with continuous acquisition. Raw data were exported and analyzed by using a custom R script applying ggPlot2 for heatmap generation.

### *In Silico* qPCR Tagging

To visualize the loxTag pattern of assembled genomes, we generated the custom script visLoxP using Python. VisLoxP creates *in silico* amplicons based on an input query sequence in fasta format and a .csv file of the oligonucleotide pairs. VisLoxP tests if a respective amplicon can be generated with user-defined requirements for the size of the amplicon and the number of accepted primer mismatches (because of not perfect long-read assemblies). If a primer pair matches the defined requirements, the value 1 is assigned if not 0. The program gives out a text file that can be further used for visualization. In this study, we used an amplicon size of ≤1000 bp and allowed ≤2 mismatches per primer. The resulting values were subsequently plotted as a heatmap using seaborn.^43^ VisLoxP is available at GitHub (https://github.com/RGSchindler/visLoxP).

### High Molecular Weight DNA Extraction and Whole Genome Nanopore Sequencing

Genomic DNA was obtained using the NucleoBond HMW DNA kit (740160.20, Macherey-Nagel, Düren, Germany) according to the manufacturer’s guidelines using lyticase (#L4025, Sigma) for cell lysis (25 μL; 10,000 U/mL) for 1 h at 37 °C in 1.5 mL of Y1 buffer (1 M sorbitol, 100 mM EDTA pH 8.0, 14 mM β-mercaptoethanol). DNA quality and concentration were assessed via a NanoDrop 8000 spectrophotometer and Qubit 3 fluorometer using dsDNA BR reagents. Library preparation was performed using the Ligation Sequencing kit SQK-LSK109 (Oxford Nanopore Technologies) with Native Barcoding kits EXP-NDB104 and EXP-NDB114 for multiplexing. Kits were used according to the manufacturers’ guidelines, except the input DNA was increased 5-fold to match the molarity expected in the protocol as no DNA shearing was applied. Sequencing was performed on a MinION Mk1B device using MinION Flow Cells [FLO-MIN111 (R10)] or Flongle Cells [FLO-FLG001 (R9.4.1)]. Table S7 lists the chemistry and flow cells used for each sample.

### Nanopore Sequencing Data Analysis

Basecalling was performed using Guppy (version: 6.0.1, Oxford Nanopore Technologies), and reads were mapped against the *S. cerevisiae* S288c reference (PRJNA128) with chromosome III replaced by synIII (PRJNA351844) using minimap2 (version 2.17-r941).^44^ Canu (version 2.2)^24^ was used to perform *de novo* assembly of linear chromosomes, and Flye (version 2.9.1-b1780)^27^ was used to assemble circular synIII derivatives. To determine the quality of the assemblies, Quast (version 5.2.0)^45^ and Inspector (version 1.0.1)^46^ were used, followed by correction based on the reference to remove potential misassemblies using RagTag (version 2.1.0).^47^ All raw reads are deposited at the NCBI Sequence Read Archive (SRA) under BioProject PRJNA884617, and an overview of sequenced strains is given in Table S1. Variant calling was performed using svim (version 2.0.0),^48^ sniffles (version 2.0.7),^49^ and Assemblatycs,^50^ and then the detected variants were merged according to its quality using SURVIVOR (version 1.0.7).^51^ Additionally, resulting variants were curated based on the coverage that supported them using samplot (version 1.3.0).^52^ Obtained synIII sequences were used to generate dot plots in comparison to the respective parental synIII using Mummer (version 3.5).^53^

### Pulsed-Field Gel Electrophoresis

Plug preparation and PFGE were performed as described previously^19^ according to methods described by Hage and Houseley^54^ with the following slight alterations: PFGE was undertaken by running samples on a 1.0% agarose gel in 0.5× TBE solution at 14 °C on a Bio-Rad clamped homogeneous electric field apparatus (CHEF-DR III, BioRad). 6 V/cm was used with 15 h switch time of 60 s followed by 15 h 120 s at 120°. The resulting gel was stained with 1× SYBR Safe (ThermoFisher Scientific) and imaged using a Typhoon RGB laser scanning system. The known karyotype BY4741 served as a size standard.

### Flow Cytometry

Ploidy of yeast cells was determined using SYTOX Green stained fixed cells in a BD Fortessa flow cytometer. Briefly, cells were grown to an OD_600_ of approximately 0.5 with 2 mL of cells collected (4 min at 2000 × *g*), washed with filtered H_2_O, and fixed in 1 mL 70% EtOH. Fixed cells were washed twice in 1 mL of filter-sterilized 50 mM sodium citrate followed by resuspension in 1 mL of 50 mM sodium citrate with RNase A (final conc. 0.25 mg/mL) and incubation at 50 °C for 1 h. Subsequently, Proteinase K was added with a final concentration of 0.4 mg/mL followed by 1 h at 50 °C before harvesting the cells (4 min at 2000 × *g*). Cell pellets were resuspended in 1 mL of 50 mM sodium citrate with SYTOX Green (5 mM stock; 1:5000 diluted) and subsequently measured in the BD Fortessa flow cytometer. Known haploid (BY4741) and diploid (BY4743) control strains were used as standards.

### Phenotyping on Solid Media

Selected strains were recovered from cryocultures and streaked on YEPD to obtain single colonies. Individual clones were grown in 5 mL of YEPD for 40 h. Cultures were measured and normalized to an OD_600_ = 1. 10-fold dilution series were prepared in 96-well microtiter plates, and the Rotor HDA+ (Singer Instruments) was used to perform 7 × 7 grit pinning on the described solid media. Plates were cultivated at 30 °C over a time course of 5 days. Images were taken every 24 h using PhenoBooth+ (Singer Instruments).

## Data Availability

Nanopore sequencing data are deposited at the NCBI Sequence Read Archive (SRA) under BioProject PRJNA884617. The L-SCRaMbLE plasmid is available at Addgene (ID #100537). All materials created within this study are available from the corresponding authors upon request.

## Supporting Information Available

LoxTag amplicon test; cell survival assay; loxTag screening; comparison of qPCR analyses; dot plots; pulsed-field gel electrophoresis; quality assessment of Nanopore sequencing data; normalized coverage plots; ploidy analysis; details of sequenced strains; yeast strains, plasmids, oligonucleotides, and synIII loxTags used in this study; references used for loxTag generation; and details of sequenced Nanopore samples (PDF)
Supporting Data S1: predicted loxTag primer pairs (XLSX)
Supporting Data S2: genBank sequences (ZIP)

## Accession Codes

Code for qTagGer is available at https://github.com/RGSchindler/qTagGer. Code for visLoxP is available at https://github.com/RGSchindler/visLoxP.
