# Supplementary material for: An Optimized Genotyping Workflow for Identifying Highly SCRaMbLEd Synthetic Yeasts
Source: ACS Synth Biol. 2024 Apr 10;13(4):1116–27. doi: 10.1021/acssynbio.3c00476 (PMC11036488; doi:10.1021/acssynbio.3c00476)
Supplement: Supplementary file 1 — sb3c00476_si_001.pdf [file sb3c00476_si_001.pdf]

Supporting Information

An optimized genotyping workflow for identifying highly SCRaMbLEd synthetic yeasts

Author List Timon A. Lindeboom[1,§], María del Carmen Sánchez Olmos[1,§], Karina Schulz[2], Cedric K. Brinkmann[1], Adán A. Ramírez Rojas[1], Lena Hochrein[2,*], Daniel Schindler[1,3,*]

[1] Max Planck Institute for Terrestrial Microbiology, Karl-von-Frisch-Str. 10, 35043 Marburg, Germany

[2] University of Potsdam, Department of Molecular Biology, Karl-Liebknecht-Str. 24/25, 14476 Potsdam, Germany

[3] Center for Synthetic Microbiology, Philipps-University Marburg, Karl-von-Frisch-Str. 14, 35032 Marburg, Germany

[§] These authors contributed equally

*** Corresponding Authors**

Lena Hochrein: hochrein@uni-potsdam.de,

Daniel Schindler: daniel.schindler@mpi-marburg.mpg.de

Content:

Supporting figures S1-S12

Supporting tables S1-S7

Supporting references

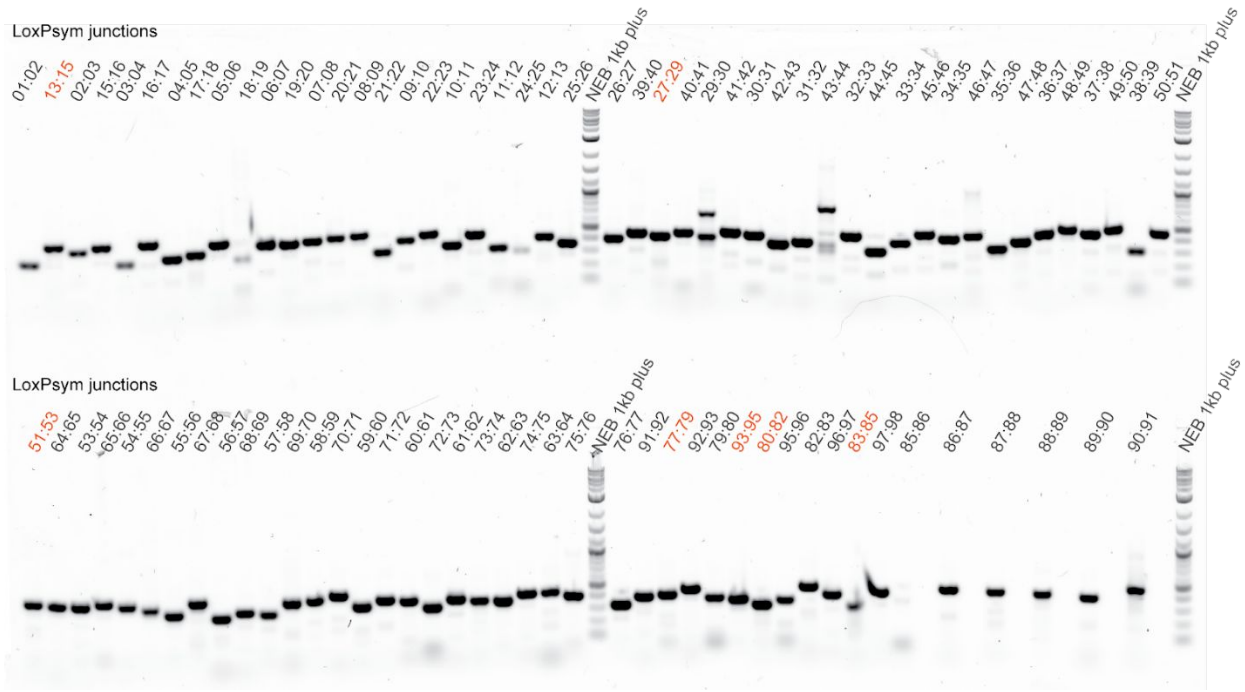

Figure S1 | LoxTag amplicon test with standard Taq polymerase. Initial test of the loxTags with standard Taq polymerase (OneTaq 2X Master Mix, NEB) and synIII genomic DNA as template. For maximal resolution and sensitivity the Typhoon RGB laser scanning system was used to identify unspecific binding. Notably, no negative control can be performed because the primer pairs can bind in both the synthetic and wild type chromosome III. Gel scan shows individual bands of the loxTags with the expected sizes (*cf*. Table S5). Amplicons spanning 2 *loxPsym* are indicated in orange, the reason for this is the small LU sizes ranging from 52 bp to 191 bp.

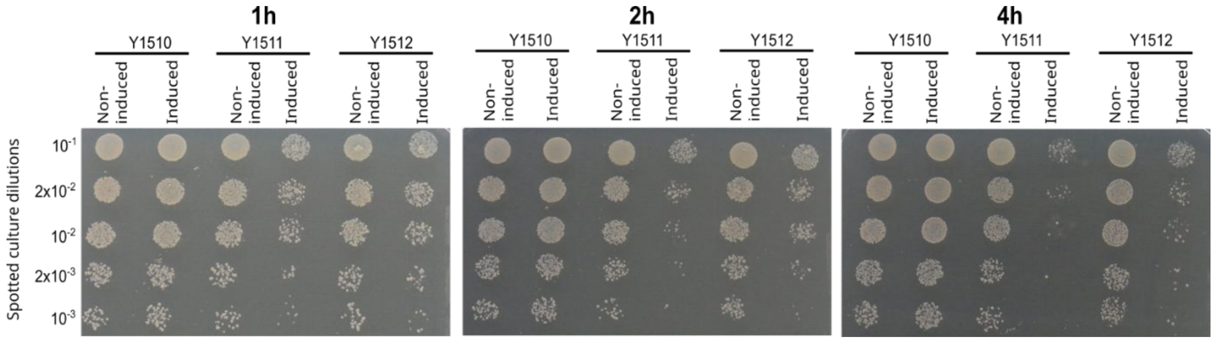

Figure S2 | Cell survival assay after red light-activated L-SCRaMbLE. In order to determine an appropriate induction time, L-SCRaMbLE was applied on wild type BY4742 (Y1510), linear synIII (Y1511) and circular synIII (Y1512), each carrying pLH_Scr15 with the expression cassettes of the red light-regulated Cre recombinase. Reduction of colony numbers results from the loss of essential genes caused by L-SCRaMbLE and is therefore a proxy for Cre activity. Y1510-Y1512 cells were grown in darkness for 6 h. Induced samples were cultured in medium containing 25 µM PCB and irradiated by a 5-min red light pulse, followed by 10-sec red light pulses every 5 min for 1 h, 2 h and 4 h, respectively. Non-induced samples were cultured in darkness for 1 h, 2 h and 4 h, respectively. After the indicated time, serial dilutions were performed and 20 µL of each sample was spotted on SC-Leu plates. Each experiment was performed in three independent biological replicates. One representative replica is shown. While no obvious difference in cell growth was observed for Y1510 as expected, the colony number decreased sharply after only one hour of red light treatment of Y1511 and Y1512.

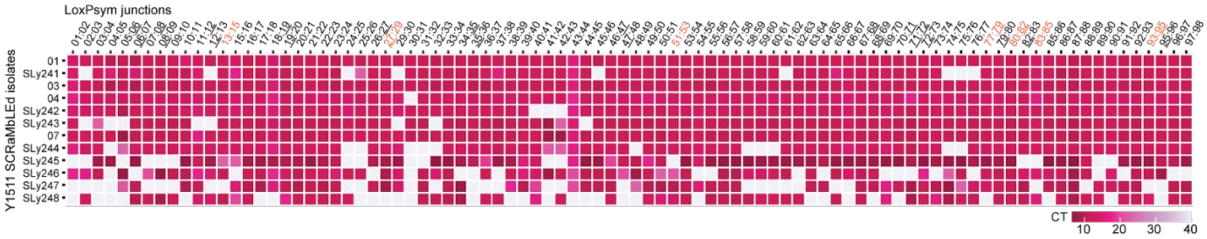

Figure S3 | LoxTag screening of L-SCRaMbLE yeast isolates obtained without selection pressure. Y1511 was induced via red light for 1 hour (10-s red light pulses applied every 5 min) and resulting cultures were plated on SC. Twelve colonies were randomly selected and analyzed by loxTag screening (initial methodology). Eight candidates were selected for long-read sequencing based on a high number of observed variations and clear differences between the selected candidates. These candidates are represented by their corresponding unique identifier (SLy241 to SLy248). The candidates with numeric identifiers are not further investigated. Orange *loxPsym* junctions indicate loxTags which span 2 *loxPsym*, essential LUs are indicated by underlined numbers. Red boxes indicate crossing of the threshold indicating the presence of amplicons. Gray boxes indicate the absence of the respective amplicon because the threshold has not been crossed.

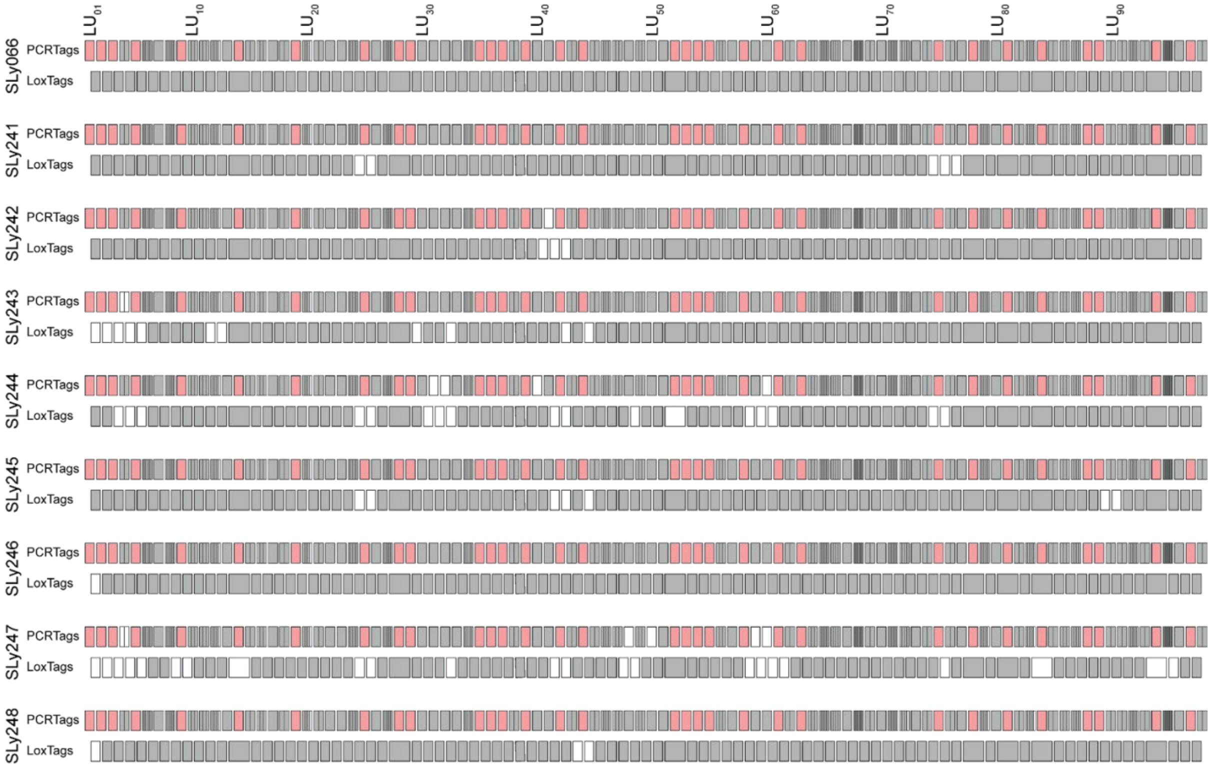

Figure S4 | Comparison of qPCR analyses using either loxTags or PCRTags. Eight SCRaMbLEd synIII strains SLy241 to SLy248 and the original synIII strain SLy066 as a reference were analyzed via qPCR with either loxTags or PCRTags following the optimized qPCR methodology. The PCR results of the reference strain SLy066 indicate that all loxTags generate an amplicon as expected. The results of the SCRaMbLEd strains show that loxTags indicate more potential SCRaMbLE events, in contrast to PCRTags as intended. Gray boxes indicate presence and white boxes absence of amplicon. LUs without PCRTags are indicated in red and loxTags spanning two LUs are indicated by larger sizes of boxes.

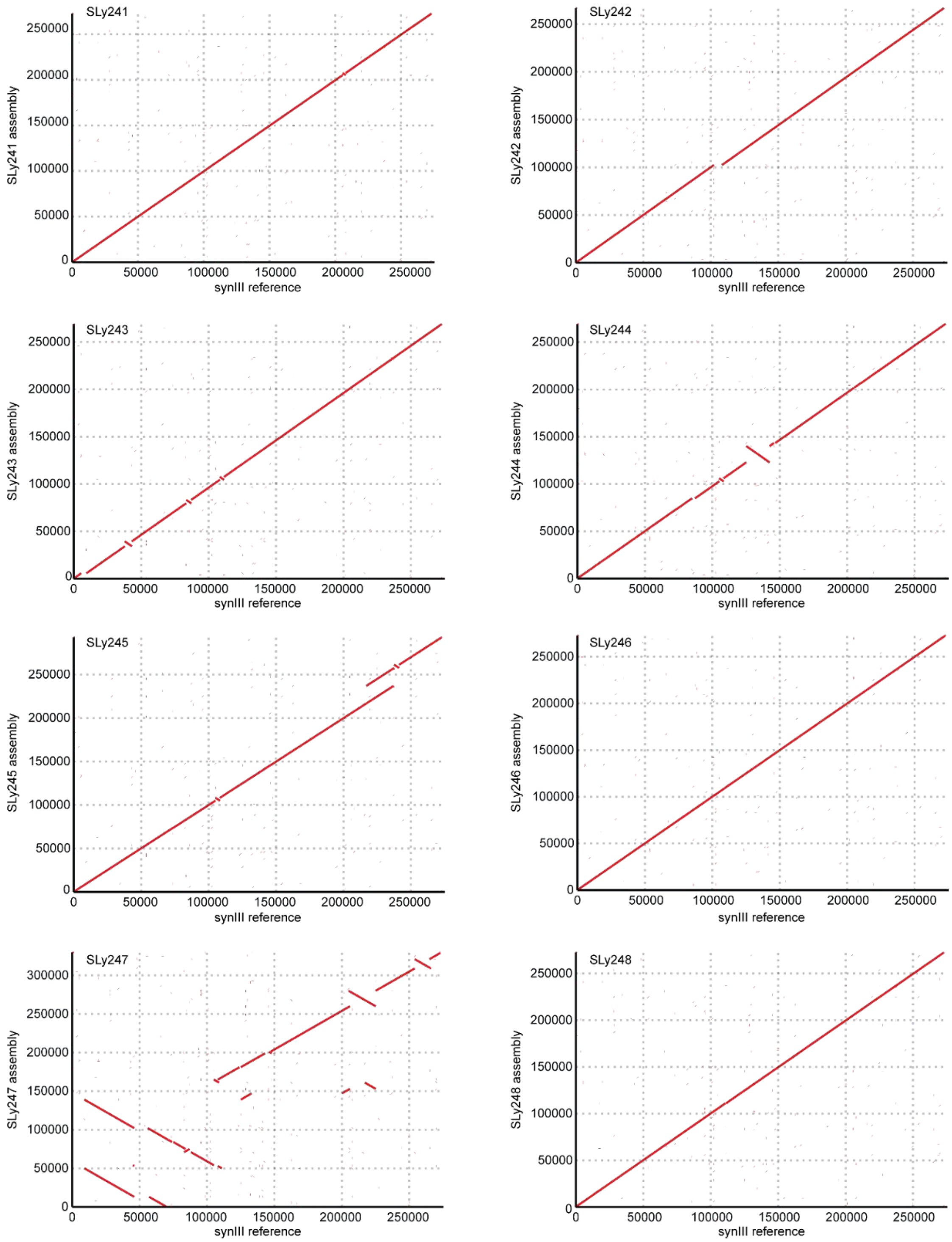

Figure S5 | Dot plots of long-read *de novo* assemblies in comparison to the parental strain. The dot plots visualize structural variations of the SCRaMbLEd isolates in comparison to the synIII reference strain SLy066. X-axis corresponds to the genomic positions of the synIII reference in bp and the y-axis corresponds to the indicated SCRaMbLEd isolate. SLy247 shows the most complex structural rearrangement including all four types of SCRaMbLE events.

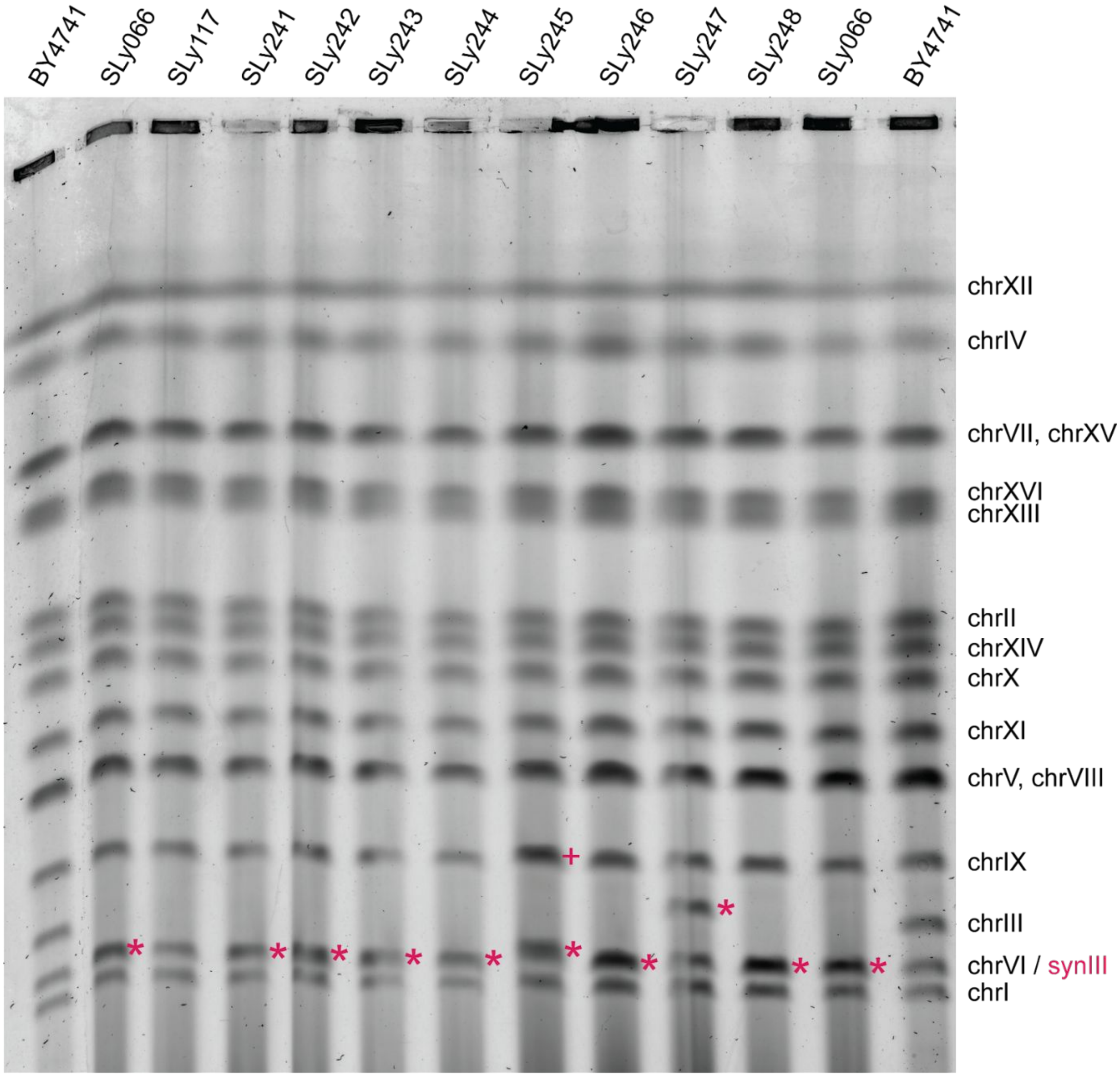

Figure S6 | Pulsed-field gel electrophoresis of the linear synIII isolates subjected to long-read sequencing. Pulsed-field gel electrophoresis was performed for wild type yeast BY4742, SLy066 (linear synIII), SLy117 (circular synIII) and SCRaMbLEd synIII strains SLy241-248. SLy245 and SLy247 show the expected size increase. SLy117 contains a circular synIII which is not visible in the analysis and serves as a control to distinguish a single chrVI band from the synIII/chrVI double band. Besides the alteration of synIII an increased intensity for chrIX in strain SLy245 is present which matches the long-read sequencing data in regard to an aneuploidy for chrIX (*cf*. Fig. 5A). * indicates synIII, + indicates chrIX aneuploidy.

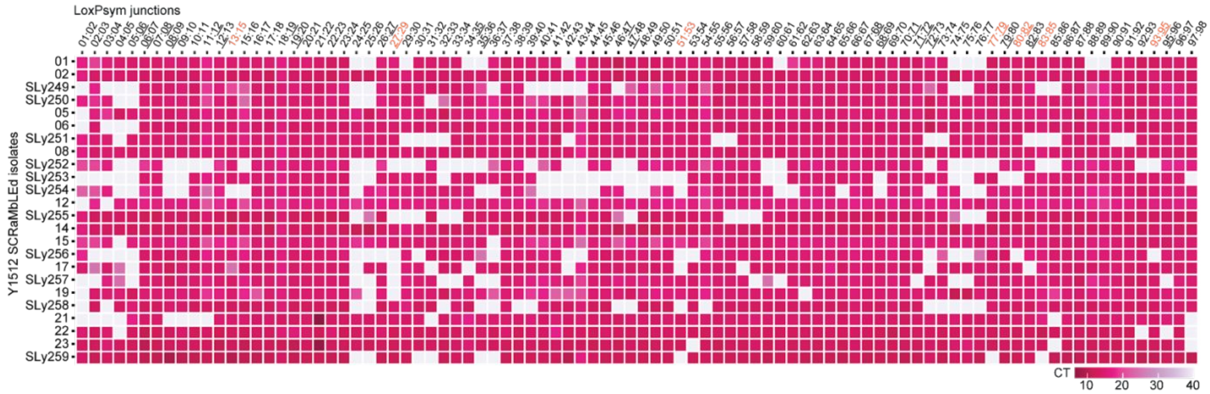

Figure S7 | LoxTag screening of L-SCRaMbLE yeast isolates with circular synIII (Y1512). Y1512 was induced via red light and plated on SC media to form single colonies. 24 colonies were randomly picked and analyzed via loxTag screening. The eleven candidates SLy249 to SLy259 were selected for long-read sequencing based on a high number of variations and clear differences between the selected candidates and are represented by their corresponding unique identifier. The candidates with numeric identifiers are not further investigated. Orange *loxPsym* junctions indicate loxTags which span 2 *loxPsym*, essential LUs are indicated by underlined numbers. Red boxes indicate qPCR samples whose fluorescence intensity has exceeded the threshold, indicating the presence of amplicons. Gray boxes indicate the absence of the respective amplicon as the threshold was not crossed.

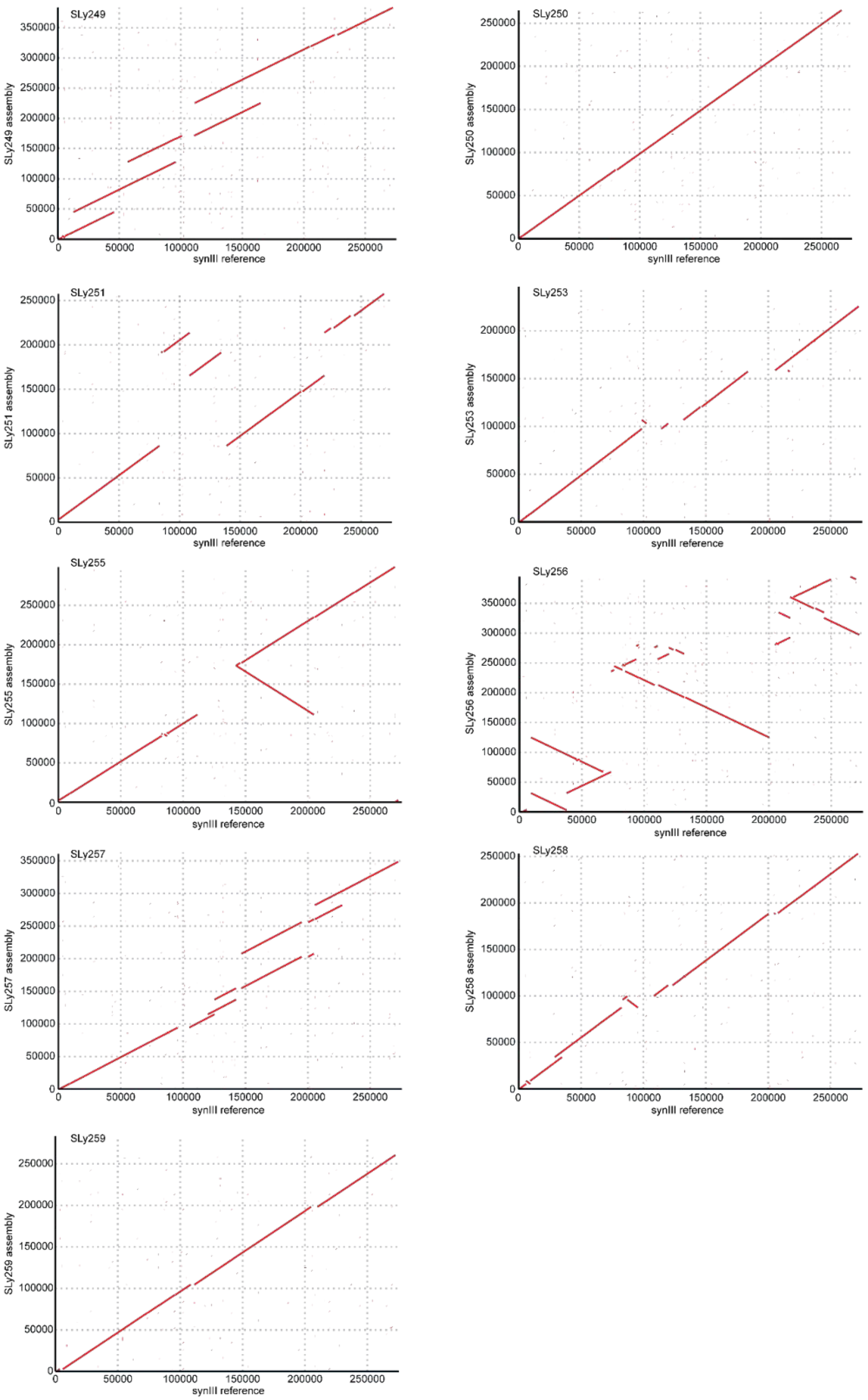
SLy249
SLy249 assembly
synIII reference
SLy250
SLy250 assembly
synIII reference
SLy251
SLy251 assembly
synIII reference
SLy253
SLy253 assembly
synIII reference
SLy255
SLy255 assembly
synIII reference
SLy256
SLy256 assembly
synIII reference
SLy257
SLy257 assembly
synIII reference
SLy258
SLy258 assembly
synIII reference
SLy259
SLy259 assembly
synIII reference

Figure S8 | Dot plots of long-read *de novo* assemblies for SCRaMbLEd circular synIII isolates SLy249-259 in comparison to the parental strain SLy117. The dot plots visualize structural variations of the SCRaMbLEd isolates in comparison to the synIII reference strain. X-axis corresponds to the genomic positions of the synIII reference and the y-axis corresponds to the synIII assembly of the indicated SCRaMbLEd isolate.

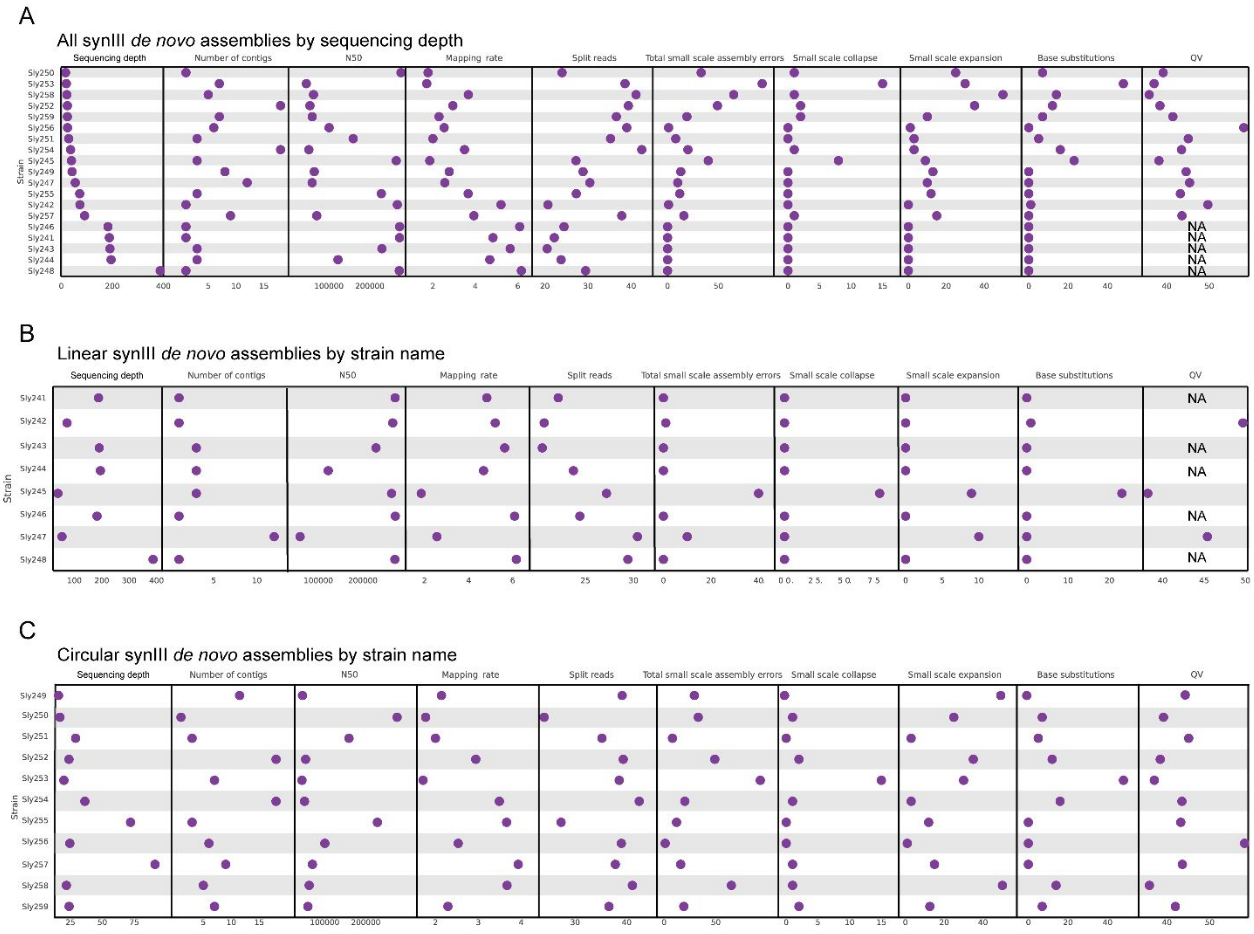

N50	Value for contiguity of an assembly. Defined by the length of the shortest contig for which longer and equal length contigs cover at least 50% of the assembly.
Mapping rate	Indicates the proportion of reads that aligne to assembled contigs. A higher mapping rate implicates better completeness of an assembly, lower mapping rate implicates parts have not been reconstructed during the assembly
Split reads	Proportion of aligned reads that have split alignments. A low rate indicates consistency between reads and assemblies and low errors. A high rate suggests that there are assembly errors causing divergence between reads and assembled contigs.
QV	Describes the assembly quality based on the identified structural and small-scale errors scaled by the total base pairs of the assembly high value indicates high quality, no value (NA) indicates it is correctly assembled.

Figure S9 | Quality assessment of Nanopore sequencing data and de novo assembly for each sample. (A) Dot chart depiction of quality scores for all synIII *de novo* assemblies sorted by sequencing depth. (B) Quality scores for *de novo* linear synIII assemblies and (C) circular synIII *de novo* assemblies sorted by strain names, respectively. In general strains with a higher sequencing depth and N50 scores show a lower number of small-scale errors. The QV value for multiple strains reflects the difficulty to solve highly SCRaMbLEd synthetic chromosome sequences with existing de novo assembly tools. This highlights the need for dedicated tools to assemble highly SCRaMbLEd synthetic yeast strains. QVs are calculated based on small-scale errors (collapse and expansion) divided by the total base pairs of the assembly.[1] Strains with no QV (NA) lack structural and small-scale error and most likely are assembled correctly.

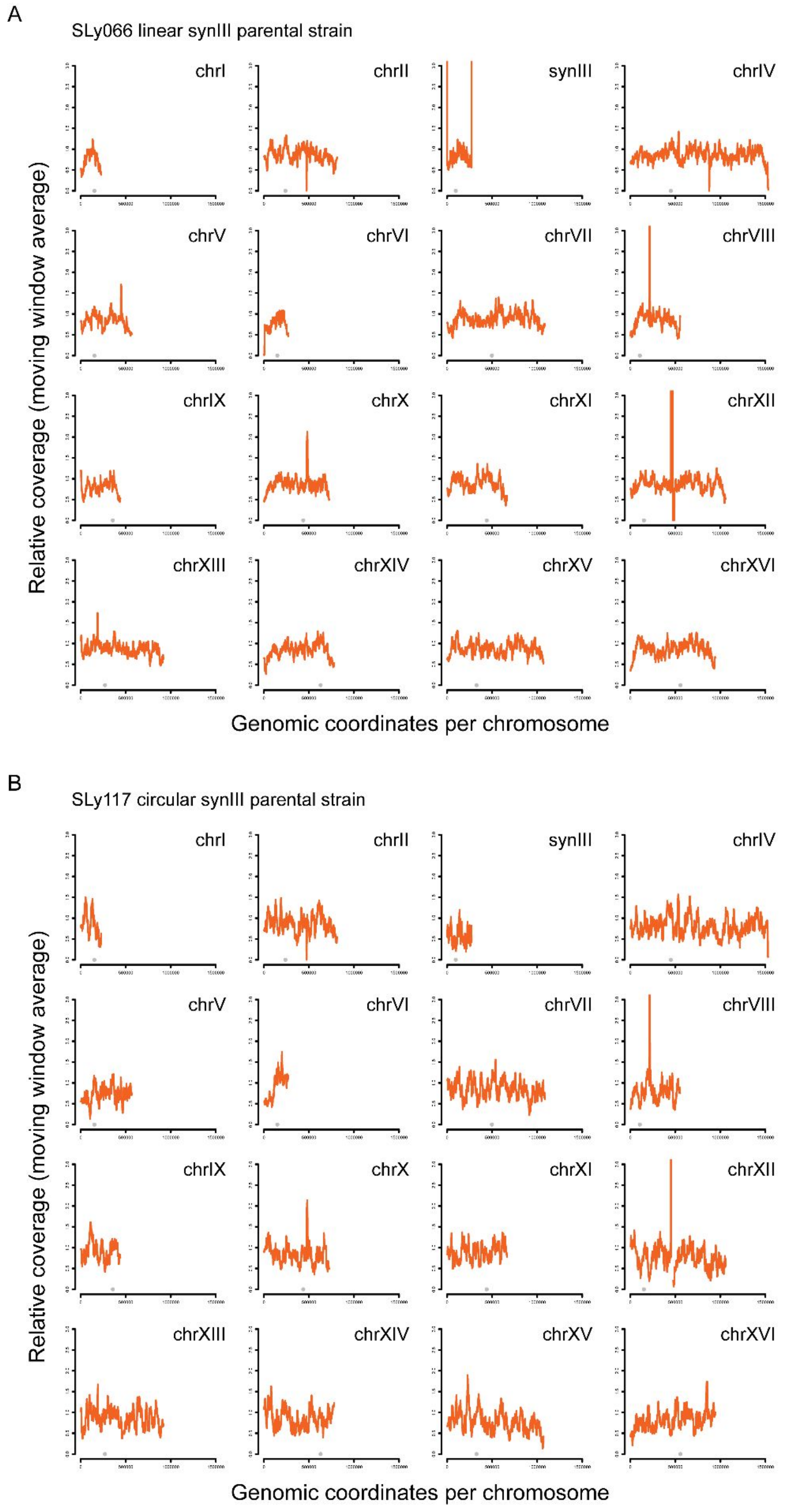

[Figure continued on the next page]

C

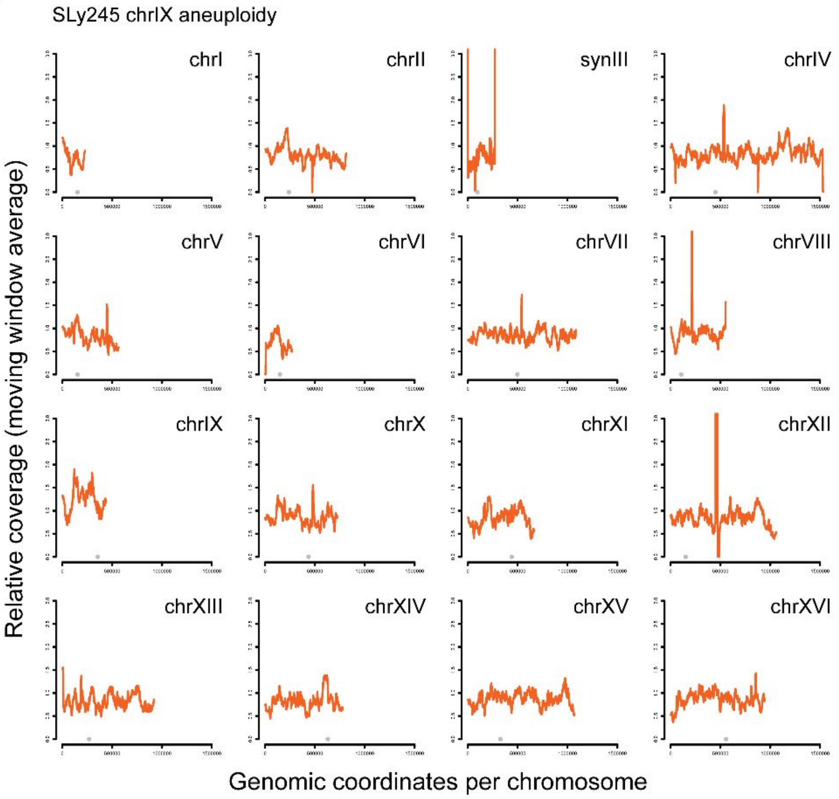

D

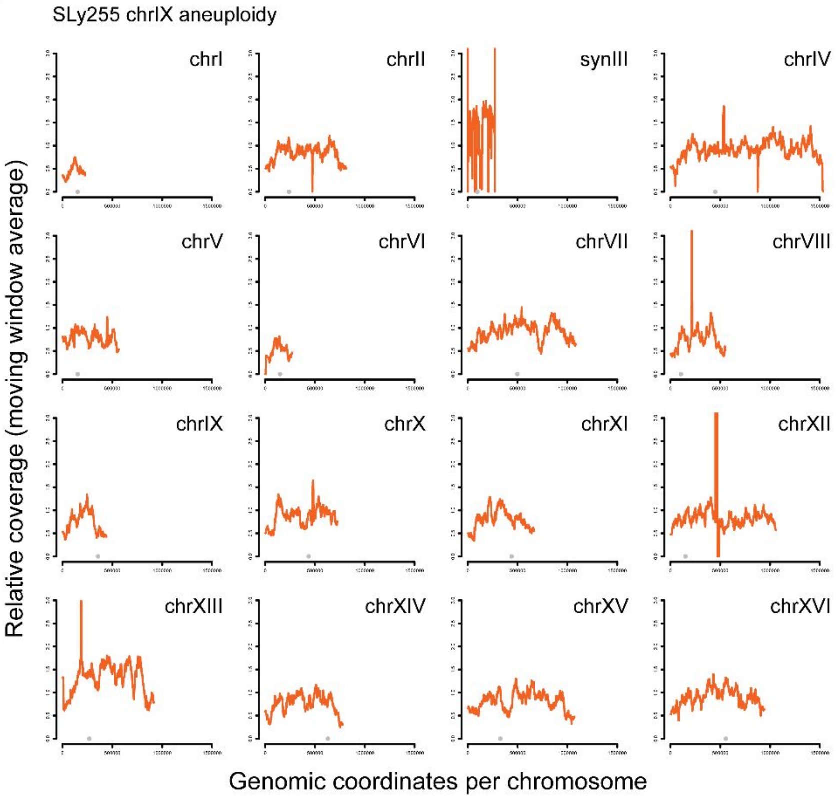

[Figure continued on the next page]

E

SLy257 chrIX aneuploidy

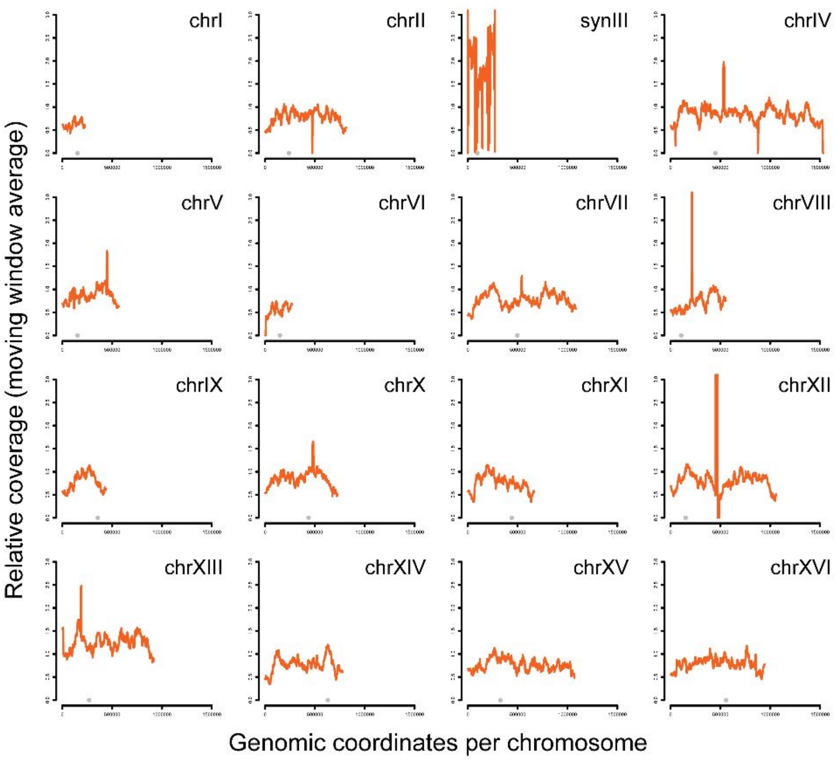

F

SLy251 segmental duplication of chrVIII

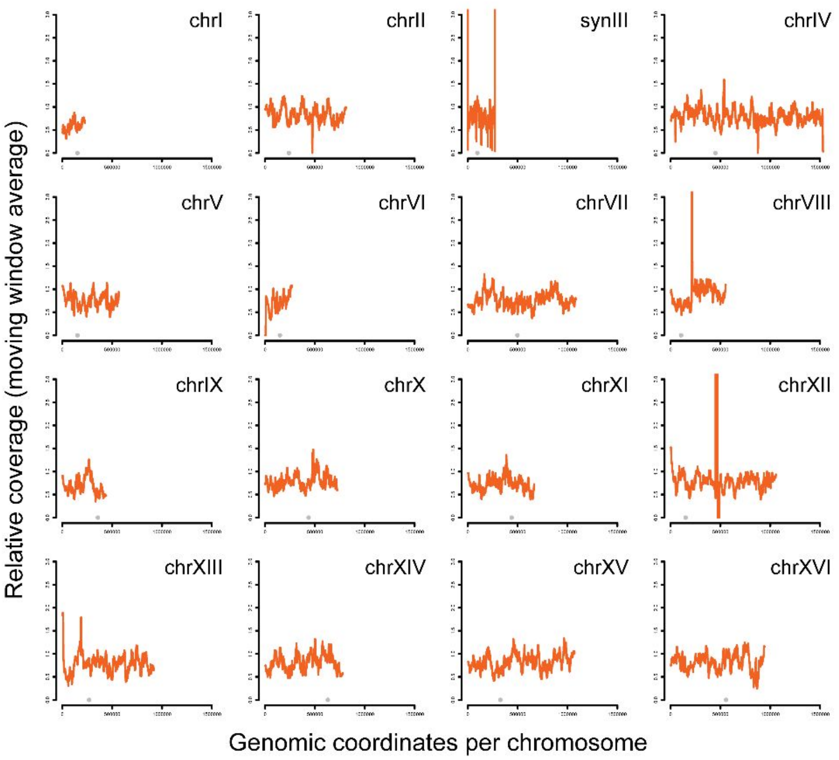

[Figure continued on the next page]

G

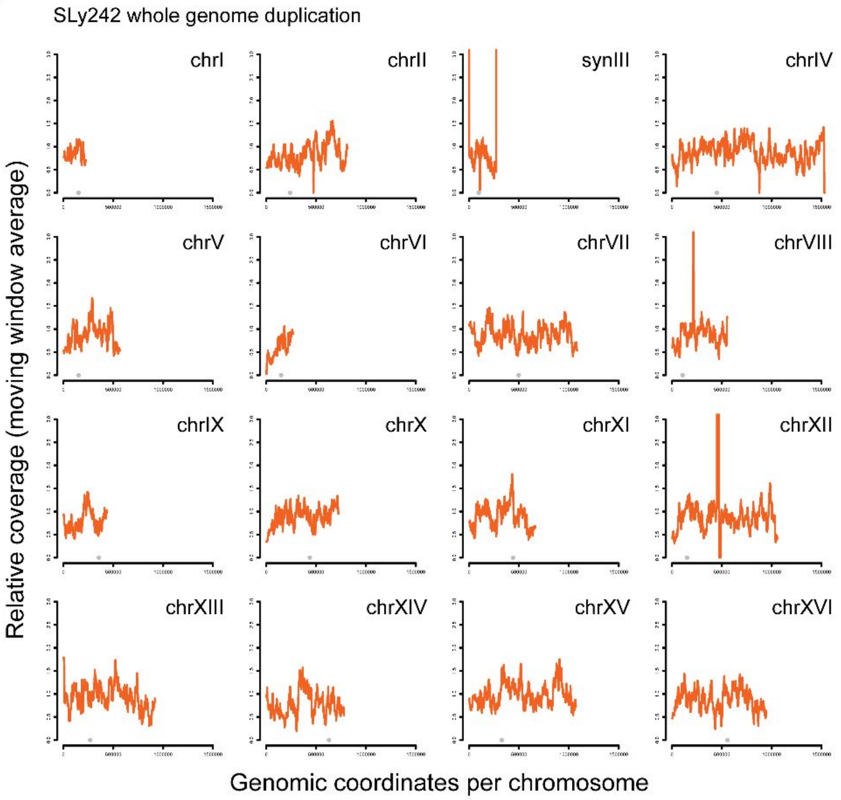

H

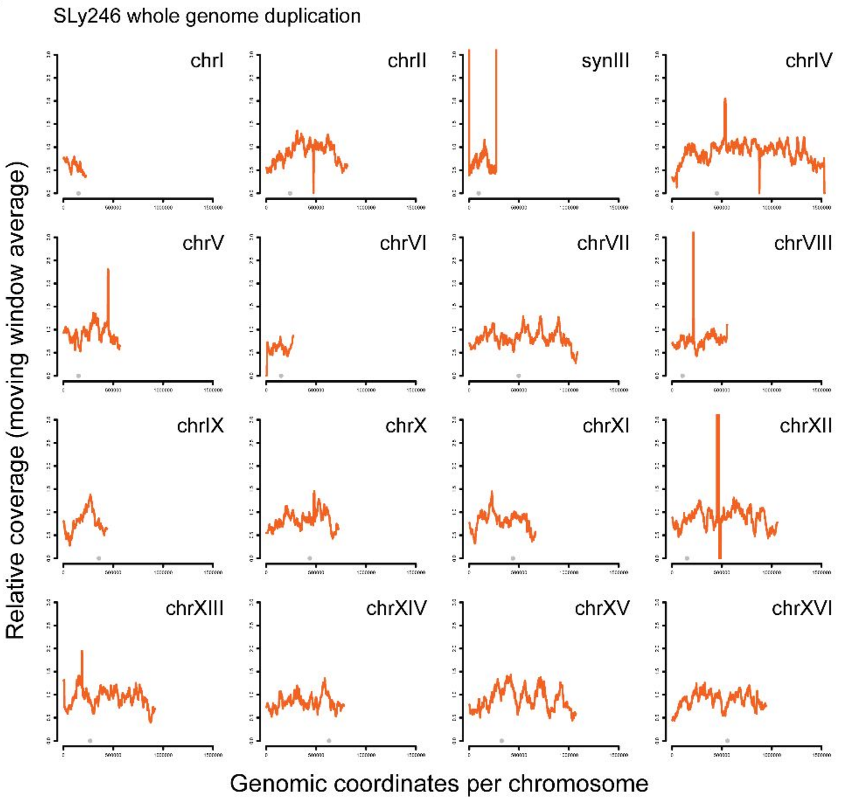

[Figure legend on the next page]

I

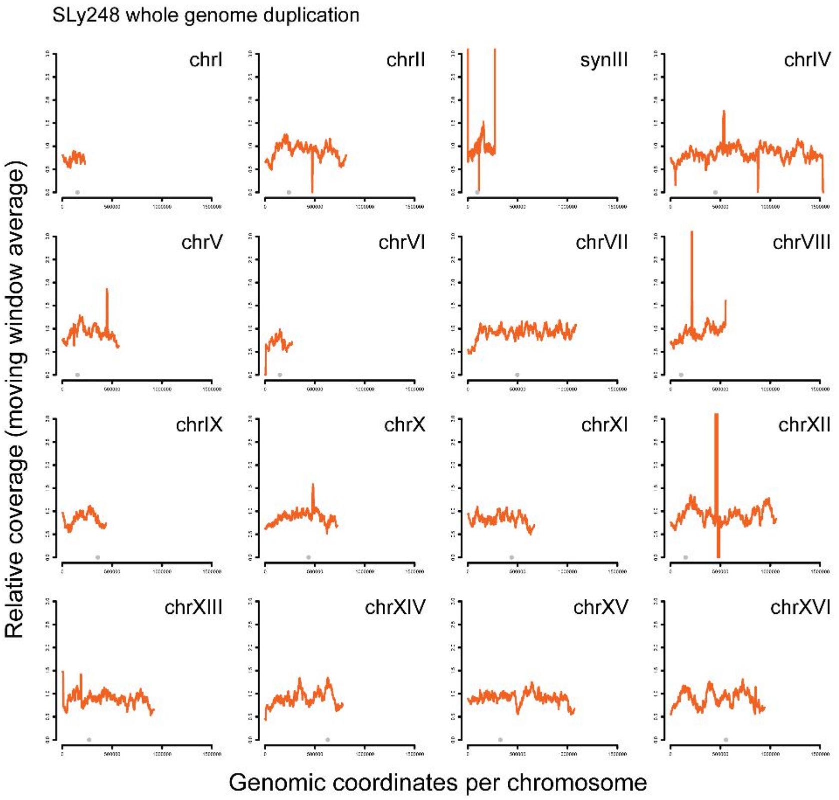

Figure S10 | Normalized coverage plots for all SCRaMbLEd strains with aneuploidy or whole genome duplication in comparison to the parental strain. (A) Linear (SLy066) and (B) circular (SLy117) synIII parental strains. The circular synIII in SLy117 shows a generally reduced abundance in comparison to the other chromosomes and the linear synIII in SLy066. SLy117 shows a potential amplification of the right arm of chrVI that is not visible in pulsed-field gel electrophoresis (see Fig. S6), and none of the progeny shows this pattern. We conclude this may be an artifact of the relatively low sequencing coverage of the strain (14-fold). Furthermore, this strain does not have a whole genome duplication (see Fig. S13). (C) to (E) strains with chrIX (SLy245), chrXIII (SLy255) and chrXIII (SLy257) aneuploidy, respectively. (F) SLy251 with segmental duplication of chrVIII. (G) to (I) Strains with whole genome duplication (SLy242, SLy246 and SLy248). X-axis and y-axis are normalized for consistent sizes. Moving window averages are plotted using 2500 bp windows and 200 bp steps, except for synIII where 100 bp windows and 20 bp steps are used. Gray dot visualizes the position of the centromere.

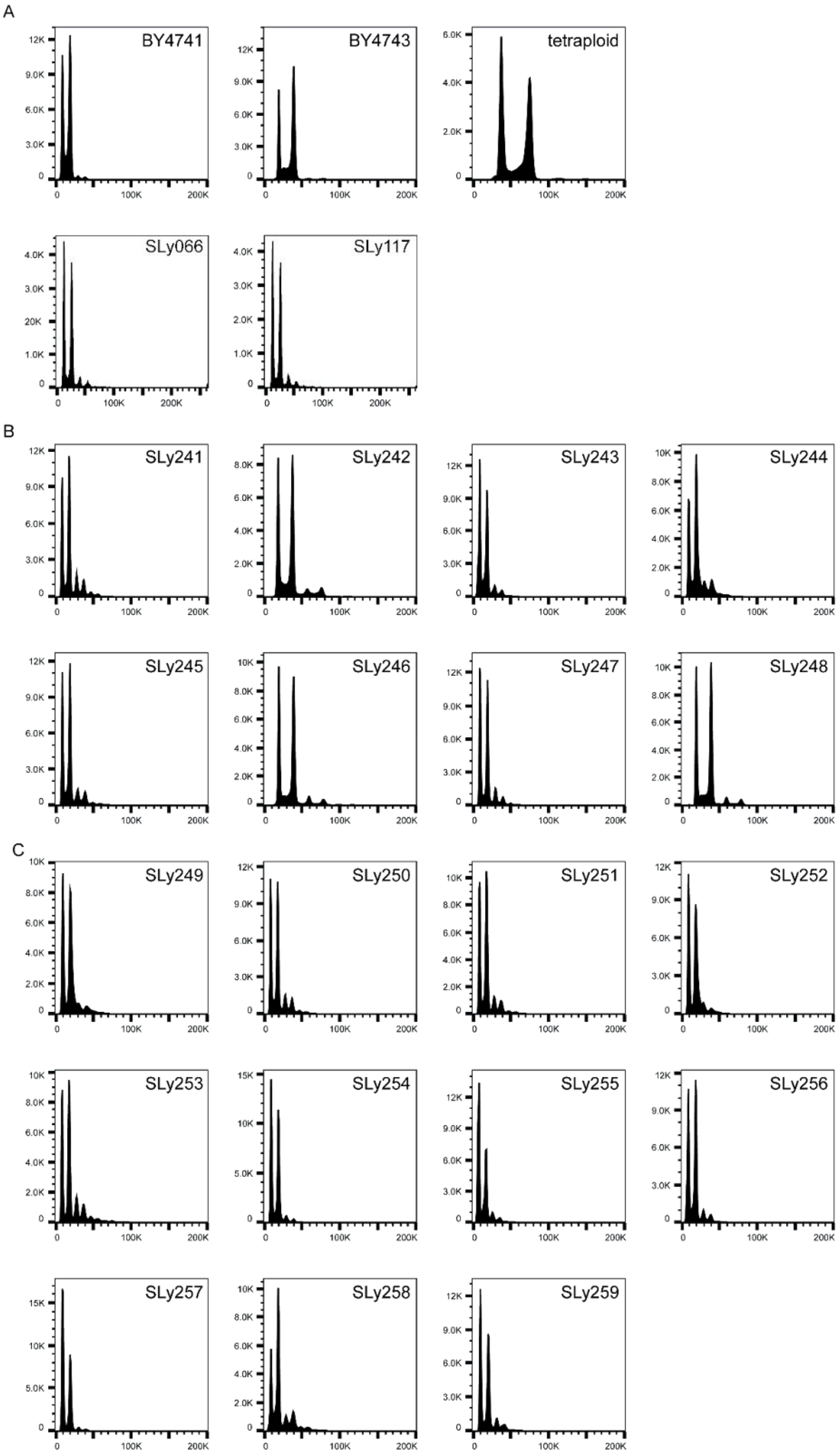
A
BY4741
BY4743
tetraploid
SLy066
SLy117
B
SLy241
SLy242
SLy243
SLy244
SLy245
SLy246
SLy247
SLy248
C
SLy249
SLy250
SLy251
SLy252
SLy253
SLy254
SLy255
SLy256
SLy257
SLy258
SLy259

Figure S11 | Ploidy analysis of SCRaMbLE isolates and reference strains by flow cytometry. (A) DNA content of control strains and parental strains (SLy066 and SLy117) used for L-SCRaMbLE. The parental strains are haploid and have no aneuploidies based on our NGS sequencing results (data not shown). Control strains visualizing known ploidies and serving as standard for the flow cytometry based DNA content determination. Haploid (BY4741), diploid (BY4743) and tetraploid (YCy2990; Schindler & Cai unpublished). Parental strains show the same DNA content as BY4741 and are consequently haploid. **(B)** Flow cytometry analysis of SCRaMbLEd linear synIII strains SLy241-248. SLy242, SLy246 and SLy248 of the linear synIII derivatives show a diploid DNA content after SCRaMbLE and indicate a whole genome duplication (no aneuploidies are detected in the NGS data). All remaining strains have a haploid genotype except for SLy245 where an aneuploidy was detected by long-read sequencing (*cf*. Fig. 5). **(C)** Flow cytometry analysis of SCRaMbLEd circular synIII strains SLy249-259. None of the circular synIII derivatives indicate a whole genome duplication, all strains are haploid. However, a partial chromosome duplication (SLy251) and aneuploidies were detected by long-read sequencing (SLy255 and SLy257; *cf*. Fig. 5).

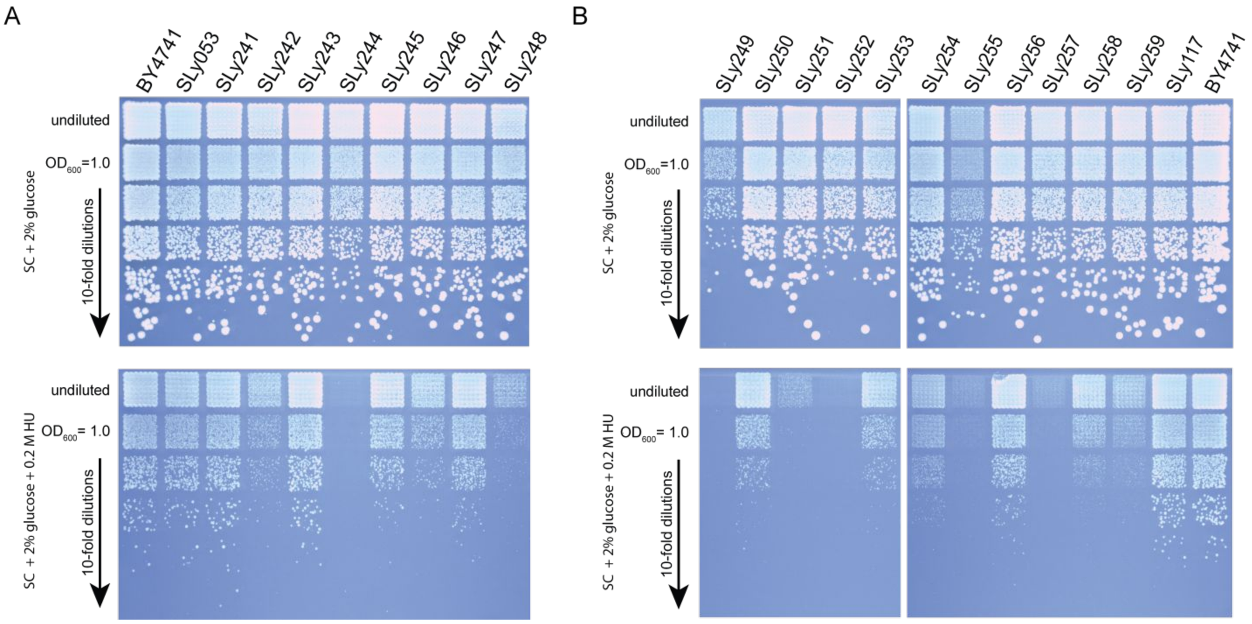

Figure S12 | Phenotypic screen for whole genome duplication based on sensitivity to hydroxyurea is not suitable to analyze SCRaMbLEd strains. (A) Linear synIII and **(B)** circular synIII isolates were tested for a growth deficiency in the presence of 0.2 M hydroxyurea (HU). The top panel shows the reference conditions, the lower panel the condition with HU. The strains SLy242, SLy246 and SLy248 showed the expected growth defect based on the observation of whole genome duplication observed by flow cytometry. The same was observed for the strains SLy255 and SLy257 containing a chromosome XIII +1 aneuploidy and SLy251 with a segmental duplication of chromosome VIII observed by whole genome sequencing. However, this was not the case for SLy245 with a duplication of chromosome IX. Besides the aforementioned strains SLy244, SLy249, SLy252 and SLy259 show different intensities of growth defects which cannot be explained by a singular cause and therefore we conclude a simple phenotypic screen on media containing HU is not suitable to screen for aneuploidies in isolates of SCRaMbLE experiments and flow cytometry should be used to determine the DNA content of relevant SCRaMbLEd isolates.

Tab. S1| Details of sequenced strains. All data are deposited at NCBI SRA under BioProject PRJNA884617.

Strain ID	Genome coverage*	Average read length	Max. read length	SRA accession
SLy066	43.94	8,495	164,359	SAMN40121705
SLy117	14.00	13,237	173,687	SAMN40121706
SLy241	44.17	17,027	319,665	SAMN32112756
SLy242	16.36	18,705	182,489	SAMN32112757
SLy243	39.75	19,254	210,066	SAMN32112758
SLy244	42.44	15,830	200,396	SAMN32112759
SLy245	33.77	20,714	215,503	SAMN32112760
SLy246	38.84	25,051	253,179	SAMN32112761
SLy247	50.05	15,783	226,520	SAMN32112762
SLy248	73.37	19,836	227,007	SAMN32112763
SLy249	36.35	18,346	215,975	SAMN32112764
SLy250	16.17	17,867	248,777	SAMN32112765
SLy251	26.14	14,486	230,528	SAMN32112766
SLy252	17.05	22,474	167,415	SAMN32112767
SLy253	18.4	21,260	209,689	SAMN32112768
SLy254	29.26	20,652	253,720	SAMN32112769
SLy255	34.77	28,742	278,058	SAMN32112770
SLy256	21.82	18,307	232,287	SAMN32112771
SLy257	56.38	25,174	291,363	SAMN32112772
SLy258	51.09	15,073	164,359	SAMN32112773
SLy259	14.65	12,089	227,073	SAMN32112774

* based on the average coverage of chromosome 1 to 16, excluding mtDNA.

Tab. S2 | Yeast strains generated and used in this study.

Strain ID	Genotype [plasmid]	Parental strain	Reference
BY4741	*MAT***a** *his3Δ1 leu2Δ0 met15Δ0 ura3Δ0*	NA	[2]
BY4742	*MAT***a** *his3Δ1 leu2Δ0 lys2Δ0 Δ0 ura3Δ0*	NA	[2]
BY4743	*MAT***a**/α *his3Δ1/his3Δ1 leu2Δ0/leu2Δ0 LYS2/lys2Δ0 met15Δ0/MET15 ura3Δ0/ura3Δ0*	BY4741 x BY4742	[2]
synIII	*MATα his3Δ1 leu2Δ0 lys2Δ0 ura3Δ0* synIII *ho::syn.SUP61-URA3*	BY4742	[3]
SLy053	*MATα his3Δ1 leu2Δ0 lys2Δ0 ΔURA3* synIII *ho::syn.SUP61-URA3*	synIII	this study
SLy066	*MATα his3Δ1 leu2Δ0 lys2Δ0 ΔURA3* synIII *ho::syn.SUP61-ΔURA3*	SLy053	this study
SLy117	*MATα his3Δ1 leu2Δ0 lys2Δ0 ΔURA3* synIII ring chromosome *ho::syn.SUP61-ΔURA3*	SLy066	this study
Y1510	BY4742 [pLH_Scr15]	BY4742	this study
Y1511	SLy066 [pLH_Scr15]	SLy066	this study
Y1512	SLy117 [pLH_Scr15]	SLy117	this study
SLy241	SLy066 SCRaMbLEd	Y1511	this study
SLy242	SLy066 SCRaMbLEd	Y1511	this study
SLy243	SLy066 SCRaMbLEd	Y1511	this study
SLy244	SLy066 SCRaMbLEd	Y1511	this study
SLy245	SLy066 SCRaMbLEd	Y1511	this study
SLy246	SLy066 SCRaMbLEd	Y1511	this study
SLy247	SLy066 SCRaMbLEd	Y1511	this study
SLy248	SLy066 SCRaMbLEd	Y1511	this study
SLy249	SLy117 SCRaMbLEd	Y1512	this study
SLy250	SLy117 SCRaMbLEd	Y1512	this study
SLy251	SLy117 SCRaMbLEd	Y1512	this study
SLy252	SLy117 SCRaMbLEd	Y1512	this study
SLy253	SLy117 SCRaMbLEd	Y1512	this study
SLy254	SLy117 SCRaMbLEd	Y1512	this study
SLy255	SLy117 SCRaMbLEd	Y1512	this study
SLy256	SLy117 SCRaMbLEd	Y1512	this study

Strain ID	Genotype [plasmid]	Parental strain	Reference
SLy257	SLy117 SCRaMbLEd	Y1512	this study
SLy258	SLy117 SCRaMbLEd	Y1512	this study
SLy259	SLy117 SCRaMbLEd	Y1512	this study
SLy273	SLy066 SCRaMbLEd	Y1426	this study
SLy274	SLy066 SCRaMbLEd	Y1426	this study
SLy275	SLy066 SCRaMbLEd	Y1426	this study

Tab. S3 | Plasmids constructed and used in this study.

Plasmid ID	Relevant features	Parental plasmid	Reference
pWS082	Guide RNA entry vector.		Addgene #90516
pWS158	Cas9 expression plasmid.		Addgene #90517
pSL0014	Used as a subcloning plasmid to amplify the circularization repair template.	pMA650 [4]	this study
pSL0096	gRNA expression vector for removal of *URA3* at *HO* locus variant 1.	pWS082	this study
pSL0097	gRNA expression vector for removal of *URA3* at *HO* locus variant 2.	pWS082	this study
pSL0098	gRNA expression vector for removal of *URA3* at *HO* locus variant 3.	pWS082	this study
pSL0270	Repair template circularization synIII.	pSL0014	this study
pSL0271	gRNA expression vector for 5’ subtelomeric region cleavage.	pWS082	this study
pSL0272	gRNA expression vector for 3’ subtelomeric region cleavage.	pWS082	this study
pSL0370	gRNA expression vector for removal of *ura3Δ0*.	pWS082	this study
pLH_Scr15	*CEN*/*ARS* plasmid with expression cassettes for PhyBNT-CreN and PIF3-NLS-CreC fusion proteins for red/far-red light-regulated Cre recombinase activity in *Saccharomyces cerevisiae*.		Addgene #100537 [5]

Tab. S4 | Oligonucleotides used in this study.

Oligo ID	Sequence (5´-> 3´)	Purpose
SLo0782	gactTTATTGGATGTTCGTACCACCAAGG	gRNA cloning to target *ura3Δ0* of chrV.
SLo0783	aaacCCTTGGTGGTACGAACATCCAATAA	gRNA cloning to target *ura3Δ0* of chrV.
SLo0804	TGGCTGTGGTTTCAGGGTCC	Forward primer 5´ *ura3Δ0* flank on chrV for creation of repair template by overlap extension PCR.
SLo0805	GATTTATCTTCGTTTCCTGCAGGTTTTTGTTCTGT	Reverse primer 5´ *ura3Δ0* flank on chrV for creation of repair template by overlap extension PCR.
SLo0806	GCAGGAAACGAAGATAAATCGGGAATCTCGGTCGTAATGATTTC	Forward primer 3´ *ura3Δ0* flank on chrV for creation of repair template by overlap extension PCR.
SLo0807	CAGATTAGAGTACAAACGCATGAAATCC	Reverse primer 3´ *ura3Δ0* flank on chrV for creation of repair template by overlap extension PCR.
SLo1244	tcaaggtgctatcttgaccg	Forward verification primer *URA3* KO at *HO* locus.
SLo1245	aaaaatgtgtatattagtttaaaaagttgtatgtaataaaagtaaaatttaatattttgg	Reverse primer 5´ *URA3* flank at *HO* for creation of repair template by overlap extension PCR.
SLo1247	cttgagggcacaaaatgtcc	Reverse verification primer *URA3* KO at *HO* locus.
SLo1256	tttgccaagaagcacgaagc	Forward primer 5´ *URA3* flank at *HO* for creation of repair template by overlap extension PCR.
SLo1257	atcctacacagggcttaagg	Reverse primer 3´ *URA3* flank at *HO* for creation of repair template by overlap extension PCR.
SLo1258	gacttttatggaagatacaaattcag	gRNA cloning to target *URA3* at *HO* locus variant 1.
SLo1259	aaacctgaatttgtatcttccataaa	gRNA cloning to target *URA3* at *HO* locus variant 1.
SLo1260	gactttcacaactcttatgaggcccg	gRNA cloning to target *URA3* at *HO* locus variant 2.
SLo1261	aaaccgggcctcataagagttgtgaa	gRNA cloning to target *URA3* at *HO* locus variant 2.
SLo1262	gactttatagaagtgaaatcatgtcg	gRNA cloning to target *URA3* at *HO* locus variant 3.
SLo1263	aaaccgacatgatttcacttctataa	gRNA cloning to target *URA3* at *HO* locus variant 3.
SLo1452	tttattacatacaactttttaaactaatatacacattttagcctctccatttgcagattg	Forward primer 3´ *URA3* flank at *HO* for creation of repair template by overlap extension PCR.
SLo2110	NNGAAGACNNTGCCttcatgagagacccggaact	Amplification of 5´ repair template of synIII subtelomeric region for subsequent Golden Gate cloning.
SLo2111	NNGAAGACNNCTTAcgcaagttcagatgtgggtc	Amplification of 5´ repair template of synIII subtelomeric region for subsequent Golden Gate cloning.
SLo2112	NNGAAGACNNtaagattcgagtctgggtgc	Amplification of 3´ repair template of synIII subtelomeric region for subsequent Golden Gate cloning.

Oligo ID	Sequence (5´-> 3´)	Purpose
SLo2113	NNGAAGACNNTCCCggcatttaggggatactgtc	Amplification of 3´ repair template of synIII subtelomeric region for subsequent Golden Gate cloning.
SLo2114	gactTTaagttatgcggccgccaccg	gRNA cloning to target the 5' subtelomeric region of synIII.
SLo2115	aaaccggtggcggccgcataacttAA	gRNA cloning to target the 5' subtelomeric region of synIII.
SLo2116	gactTTattcaaaaccgattaatagg	gRNA cloning to target the 3' subtelomeric region of synIII.
SLo2117	aaaccctattaatcggttttgaatAA	gRNA cloning to target the 3' subtelomeric region of synIII.
SLo2118	tctttaccgtcatgcacgtg	Forward verification primer of synIII circularization.
SLo2119	ctcacccgaaagagatgctg	Reverse verification primer of synIII circularization.

Tab. S5 | synIII loxTags used in this study. All loxTags (incl. synIII) and the roxTags are provided with the supporting data (Supporting Data S1).

LoxPsym junction*	Oligo ID	FWD	Oligo ID	REV
LU 01:02	SLo3432	TGCCACATTTCCTTTGCAGC	SLo3522	GCGTCTGAACCACATGCTTC
LU 02:03	SLo3433	CTACGACGGTTCTCATTCGAC	SLo3523	TTCCAAATAAGTCCGTGCCG
LU 03:04	SLo3434	TTCGGGGGCCAAATCTAACC	SLo3524	TTCTAGGCCGGCAATGCAC
LU 04:05	SLo3435	GAGGGGGAGAAAGAATAAGGGC	SLo3525	AGATGGCACCCGAGTCTAGG
LU 05:06	SLo3436	CTTGGCCTAGACTCGGGTG	SLo3526	AAAGCGCCCAGAGGATGAAG
LU 06:07	SLo3437	GGCGGAAGTCTTTGCAACTG	SLo3527	GGATGGGGAGGGAAATGGTC
LU 07:08	SLo3438	GGCTGGGTTATAGTGAGCCC	SLo3528	TGGCTGAAAAAGTGAACACGC
LU 08:09	SLo3439	TGTTAAGAGCTGCACCCCAG	SLo3529	GCCGTTAAGGTTACGATTCACC
LU 09:10	SLo3440	GGTGAATCGTAACCTTAACGGC	SLo3530	TCTGTGTTGTCCCTCGAAGC
LU 10:11	SLo3441	CAAGCATGCAATTGGACCCC	SLo3531	ATGGGGCGCTAGGATTCATG
LU 11:12	SLo3442	TATGGGGGAAGGGGAGGATG	SLo3532	GCCCTCCCATACCAAGACAC
LU 12:13	SLo3443	CTAGGCGCTGAAGGTGAGAG	SLo3533	GTAGTACGCCCCCTTGGAAG
LU 13:15	SLo3444	GTTTGCTTCTGGCGGTGATG	SLo3534	GATTGAGCTTGGGCGGTTTG
LU 15:16	SLo3445	AGCTTTGCTGGAGGATGATTAAC	SLo3535	CGCTACCGTCAATGTTGCAG
LU 16:17	SLo3446	ACGAGAGCCTAAAGTGGTGC	SLo3536	AGGAAGGCGCAGACATTCAG
LU 17:18	SLo3447	GCGATGAAAGTGATCACGGC	SLo3537	GAAATCTGCTGCGCTCGATG
LU 18:19	SLo3448	AAGAAGACTCCTGCGAACGG	SLo3538	TGATGATGTGCATGACAACTGC
LU 19:20	SLo3449	GTTCGTTCGCCCTAAATGCC	SLo3539	AGGAGGGTCTAGACGGTCAC
LU 20:21	SLo3450	TTAGCGCCCTAGCATCCATG	SLo3540	CGTCAAAAAGGGCATCCTGC
LU 21:22	SLo3451	TTGTGCCTGGTGACTGTCTC	SLo3541	CAACTCGTGGGTTGCATTGG
LU 22:23	SLo3452	GGAGCGGGTCTGGTGAATAG	SLo3542	TCCAACTGCCTTGCCAGAAG
LU 23:24	SLo3453	ATGCACGACGAAAACGGTTG	SLo3543	CAGGGCAGACAAGATCGACC
LU 24:25	SLo3454	ACCAAATGGGGACGGTAGAAG	SLo3544	TCTGAGATTGTTGACCGGTCC
LU 25:26	SLo3455	TGGACCGGTCAACAATCTCAG	SLo3545	CTTTTTGTGTGGTGCCCTCC
LU 26:27	SLo3456	GCAGTTCCTTCGTGTATGCG	SLo3546	GGCAAAAAGACCGTGGTCAC
LU 27:29	SLo3457	GATGGGAAACAGAACTGCGC	SLo3547	AGCAACGACGTAAGAGGTGG
LU 29:30	SLo3458	AGGTCATGGTTCGGTTGGTG	SLo3548	AAGGCGTGAGGACCTTGAAG
LU 30:31	SLo3459	GCTCTCTCTCCATCTTGGCG	SLo3549	CCCGCCAGGTACTAAGGTTC
LU 31:32	SLo3460	AGTAACGCGTCACCTCGTAC	SLo3550	ACTTTGGAGGTCGTGTCGTC
LU 32:33	SLo3461	CTGTGCATCTGCTTGTACGC	SLo3551	ATACAACCTCGTTGCCCAGG
LU 33:34	SLo3462	AGTGGTTAGTGCCATGTCCG	SLo3552	GCGTCTCGTTTCAAAGACCG
LU 34:35	SLo3463	GAGCTACCGTTATCAGCTTGC	SLo3553	GGAGGCATCTCAAGCTCTCC
LU 35:36	SLo3464	AAACCGAACCCTTCCCCTTC	SLo3554	AGCAAAACTTCCACCAGTAAACG
LU 36:37	SLo3465	AAACATGGCATGGCGATCAG	SLo3555	CGCGGACATTAAGGACGAAC
LU 37:38	SLo3466	CGCCAATCCAGCAATACCTG	SLo3556	CCGTTTGCAGTGGTTGGTTC
LU 38:39	SLo3467	GCCGAGACAAGAGGAGGAAC	SLo3557	TTCTTTCTGTTTCCGCTCCTTG

LoxPsym junction*	Oligo ID	FWD	Oligo ID	REV
LU 39:40	SLo3468	ACAAGGAGCGGAAACAGAAAG	SLo3558	CAAAAGAAGCCAAGCCCTCC
LU 40:41	SLo3469	AGTTCCAAGAAGGGGAAGCG	SLo3559	CGGTGCTTCCATTGAAAGGC
LU 41:42	SLo3470	CAGCCATCTGAGGAACCTCC	SLo3560	GGGAACAACTTCGCTTGCAG
LU 42:43	SLo3471	CTCGTGGTGAGGAGTGTTCC	SLo3561	CGGAAAATGCGTGAGATGGC
LU 43:44	SLo3472	GGCGCGCATTGAAATAAACG	SLo3562	CGATAATAACCGCGGGCATG
LU 44:45	SLo3473	CATGCCCGCGGTTATTATCG	SLo3563	GGTTACACTCGTTTAGCGCAG
LU 45:46	SLo3474	CCCTTGTTTGGCTTGCTCAC	SLo3564	ACGTGGCCTTCAAGTACCTG
LU 46:47	SLo3475	GTCTCTACTGGTGGTGGTGC	SLo3565	TATTGGCAGCGCAAAAAGGC
LU 57:48	SLo3476	GAAGGAACCGACTCAAACCG	SLo3566	GCTATAGCAACAGCATCGGC
LU 48:49	SLo3477	AGGTACAAGACGAGCAAGAGG	SLo3567	TTTAGGCGAAGGTGAGAGGG
LU 49:50	SLo3478	AGAACTGCAAACCTGAACCAAG	SLo3568	TTCAATGGCGTAGTGGACCC
LU 50:51	SLo3479	TGACTGAACCCTTACCGCTG	SLo3569	ACTGAGATTCCGATGACCGTG
LU 51:53	SLo3480	AGCTTTTTGGGTGGTTTCGC	SLo3570	CCGTCGAAGAAGTGGAAAGC
LU 53:54	SLo3481	ACGGTCCTTCAATGTTCCGG	SLo3571	GAGGAACGTATGGGCTCCAG
LU 54:55	SLo3482	GAACTCATGCACTTCGCGAG	SLo3572	AGCTTACAATTGTGAATGTTGCG
LU 55:56	SLo3483	AAAGTGGTGTTTGCGTCTCC	SLo3573	AACCCGAACCTGAAGCAGAG
LU 56:57	SLo3484	ACATAGGTCAGGCCGAATGC	SLo3574	CACGGAGGCTTTGGTCTAGG
LU 57:58	SLo3485	TTCAACTTGAGCACGCACAC	SLo3575	GGTCGGTTTGGCTTGTATTGC
LU 58:59	SLo3486	GACCCCACTGTCATCGGATG	SLo3576	TAGCCGCTATGCCATCTTCG
LU 59:60	SLo3487	GCGTCCAAGCCATTGAAAGG	SLo3577	GGTGAAAAGTTGGCCTCGAC
LU 60:61	SLo3488	GCCGTGCCCCAATAAAACTG	SLo3578	TCATTGGGAGATCAGTCGCG
LU 61:62	SLo3489	CGTGAACGCATAGCAGAGAAC	SLo3579	TGACGATGACGACAACCAGG
LU 62:63	SLo3490	CGGACTGCATGGATCAATGG	SLo3580	AGAAGATGCTGAGGGCCAAG
LU 63:64	SLo3491	TTTTTCGTCTGGGCGCAAAC	SLo3581	CGGATAACGTGGCTCTACCC
LU 64:65	SLo3492	AAAGGGGTAGCCTGCTTCAC	SLo3582	CCCCAGTTCCATCTGACTCC
LU 65:66	SLo3493	ACCTGTCCCAGAAAAGACGC	SLo3583	TGACGGCTTCTTTCCCACTG
LU 66:67	SLo3494	AGCACAGCTCGAAGACACTC	SLo3584	ATGTATTGCCCGTCTCAGCC
LU 67:68	SLo3495	TAGAGTGGTAAAGCGTGCCC	SLo3585	AACATTAGTGGGAGGAGGCG
LU 68:69	SLo3496	AGCTGGGATCATCCTTTGCC	SLo3586	TGCACAATATGGCCCTCGTG
LU 69:70	SLo3497	GAGATGGGTGGCCACAGAAC	SLo3587	ATCGAAAGCTTGGACCCAGG
LU 70:71	SLo3498	CAGCTGCTCAGAGGGAAGAC	SLo3588	GTGTGACCATGGACGAGGAG
LU 71:72	SLo3499	CTATCTACCGCTCACCCAGC	SLo3589	AAGAAGACCCTGGGAACGAG
LU 72:73	SLo3500	CCTTGCCTGTAGACCGTACC	SLo3590	AGAGAAGCTGTTGTCAGGGC
LU 73:74	SLo3501	CATGGCGCTGATCAAACTGG	SLo3591	GTTGACGCGCGAAGAAAAAC
LU 74:75	SLo3502	TACGCCAAGCTGCTCTACTG	SLo3592	AGGTGCAGGCTTGGATCTTC
LU 5:76	SLo3503	AGTCCAATGAGCAGCTCTGG	SLo3593	GGCCAGAACAATACTGCAACG
LU 76:77	SLo3504	AAGAAAGTCCTCTGCACCCG	SLo3594	GACTCATCCAACCGTTTGCG
LU 77:79	SLo3505	ACCGAGGATCAGCAAATGGAG	SLo3595	ACTACGCTGGCCTTCATGAC

LoxPsym junction*	Oligo ID	FWD	Oligo ID	REV
LU 79:80	SLo3506	GAAACTGGCTTGGCTTCTGC	SLo3596	AGTACAGGCAGAGCAGCAAG
LU 80:82	SLo3507	ACCACCCAATTTCCAGAGCC	SLo3597	AAGGGCAATGCAGTAGAGGC
LU 82:83	SLo3508	GTGGAGGAATCAGGGTGGAC	SLo3598	CGTTGCTGCAGGTCGAATAC
LU 83:85	SLo3509	TGACGACGATGCTCTTACGG	SLo3599	AAGCAGAGGTCACGACTTGG
LU 85:86	SLo3510	TCTGTATGGCGTGACACCAC	SLo3600	TATGGGGTTGGTGTATCGCG
LU 86:87	SLo3511	TCCTGGGGACTGAAAAAGGC	SLo3601	GGGGGAGTCAGCAAGTATCG
LU 87:88	SLo3512	TTTCCATGGAGCCGTACCAC	SLo3602	GAAACTTGGCCACAGCATCG
LU 88:89	SLo3513	TGGCCCCTGTAAGATGATGC	SLo3603	CCCTTCCCCTCCTTTTCCTG
LU 89:90	SLo3514	CAGGAAAAGGAGGGGAAGGG	SLo3604	TCCGGTTCCAAAGATCGTGG
LU 90:91	SLo3515	TAAACAGCGATCCACCGAGG	SLo3605	GAAGCAGAAGCAGAAGCAGC
LU 91:92	SLo3516	CACAGCTGCCATCAAGAAGC	SLo3606	ACTGAAAGGGAAGGCTCGTG
LU 92:93	SLo3517	AGTACGTATGCTGGTTCGGC	SLo3607	CCTGTGCGCTTTTGAGGATG
LU 93:95	SLo3518	CGACACAATTCTAGCCACCG	SLo3608	CAGCAACGGATAAACTCGCG
LU 95:96	SLo3519	CTCATCGTTGCAGGGCTTTG	SLo3609	TGGGAATAGCAAACCCTGGG
LU 96:97	SLo3520	ACAAGCAAGTGGGGTAACTTAG	SLo3610	TTCCCGCTACAAAGTTGTGC
LU 97:98	SLo3521	CTCTTGGGAGTAAGCTGCATC	SLo3611	AGGCGTCGAATGTTTCCAAC

* primer pairs spanning two *loxPsym* sites are indicated in orange

Tab. S6| References used for loxTag generation.

Chromosome	Reference
synI	chr01_3_24
synII	chr02_3_26
synIII	chr03_9_02
synIV	chr04_3_71
synV	chr05_3_43
synVI	chr06_3_27
synVII	chr07_3_60
synVIII	chr08_3_35
synIX	chr09_3_54
synX	chr10_9_01
synXI	chr11_3_38
synXII	chr12_9_05
synXIII	chr13_3_43
synXIV	chr14_3_29
synXV	chr15_3_45
synXVI	chr16_3_43
tRNA neochromosome	PRJNA351844

Tab. S7| Details of sequenced Nanopore samples.

Strain	Sequencing run	Sample	Flow_cell_type	Multiplexed	Library_prep_kit
SLy066	ONTrun_078	ONTrun_078_S01	FLO-FLG001 (R9.4.1)	yes	SQK-LSK109
SLy117	ONTrun_081	ONTrun_081_S01	FLO-FLG001 (R9.4.1)	no	SQK-LSK109
SLy241	ONTrun_073	ONTrun_073_S01	FLO-MIN111 (R10.1)	yes	SQK-LSK109
SLy242	ONTrun_073	ONTrun_073_S02	FLO-MIN111 (R10.1)	yes	SQK-LSK109
SLy243	ONTrun_073	ONTrun_073_S03	FLO-MIN111 (R10.1)	yes	SQK-LSK109
SLy244	ONTrun_073	ONTrun_073_S04	FLO-MIN111 (R10.1)	yes	SQK-LSK109
SLy245	ONTrun_073	ONTrun_073_S05	FLO-MIN111 (R10.1)	yes	SQK-LSK109
SLy246	ONTrun_073	ONTrun_073_S06	FLO-MIN111 (R10.1)	yes	SQK-LSK109
SLy247	ONTrun_073	ONTrun_073_S07	FLO-MIN111 (R10.1)	yes	SQK-LSK109
SLy248	ONTrun_073	ONTrun_073_S08	FLO-MIN111 (R10.1)	yes	SQK-LSK109
SLy249	ONTrun_073	ONTrun_073_S09	FLO-MIN111 (R10.1)	yes	SQK-LSK109
SLy250	ONTrun_073	ONTrun_073_S10	FLO-MIN111 (R10.1)	yes	SQK-LSK109
SLy251	ONTrun_073	ONTrun_073_S11	FLO-MIN111 (R10.1)	yes	SQK-LSK109
SLy252	ONTrun_073	ONTrun_073_S12	FLO-MIN111 (R10.1)	yes	SQK-LSK109
SLy253	ONTrun_073	ONTrun_073_S13	FLO-MIN111 (R10.1)	yes	SQK-LSK109
SLy254	ONTrun_073	ONTrun_073_S14	FLO-MIN111 (R10.1)	yes	SQK-LSK109
SLy255	ONTrun_073	ONTrun_073_S15	FLO-MIN111 (R10.1)	yes	SQK-LSK109
SLy256	ONTrun_073	ONTrun_073_S16	FLO-MIN111 (R10.1)	yes	SQK-LSK109
SLy257	ONTrun_073	ONTrun_073_S17	FLO-MIN111 (R10.1)	yes	SQK-LSK109
SLy258	ONTrun_073<br>ONTrun_078	ONTrun_073_S18<br>ONTrun_078_S02	FLO-MIN111 (R10.1)<br>FLO-FLG001 (R9.4.1)	Yes<br>yes	SQK-LSK109
SLy259	ONTrun_073	ONTrun_073_S19	FLO-MIN111 (R10.1)	yes	SQK-LSK109

Supporting references

1. Chen, Y.; Zhang, Y.; Wang, A. Y.; Gao, M.; Chong, Z., Accurate long-read *de novo* assembly evaluation with Inspector. *Genome Biol* **2021,** *22* (1), 312.
2. Brachmann, C. B.; Davies, A.; Cost, G. J.; Caputo, E.; Li, J.; Hieter, P.; Boeke, J. D., Designer deletion strains derived from *Saccharomyces cerevisiae S288C*: A useful set of strains and plasmids for PCR-mediated gene disruption and other applications. *Yeast* **1998,** *14* (2), 115-32.
3. Annaluru, N.; Muller, H.; Mitchell, L. A.; Ramalingam, S.; Stracquadanio, G.; Richardson, S. M.; Dymond, J. S.; Kuang, Z.; Scheifele, L. Z.; Cooper, E. M.; Cai, Y.; Zeller, K.; Agmon, N.; Han, J. S.; Hadjithomas, M.; Tullman, J.; Caravelli, K.; Cirelli, K.; Guo, Z.; London, V.; Yeluru, A.; Murugan, S.; Kandavelou, K.; Agier, N.; Fischer, G.; Yang, K.; Martin, J. A.; Bilgel, M.; Bohutski, P.; Boulier, K. M.; Capaldo, B. J.; Chang, J.; Charoen, K.; Choi, W. J.; Deng, P.; DiCarlo, J. E.; Doong, J.; Dunn, J.; Feinberg, J. I.; Fernandez, C.; Floria, C. E.; Gladowski, D.; Hadidi, P.; Ishizuka, I.; Jabbari, J.; Lau, C. Y.; Lee, P. A.; Li, S.; Lin, D.; Linder, M. E.; Ling, J.; Liu, J.; Liu, J.; London, M.; Ma, H.; Mao, J.; McDade, J. E.; McMillan, A.; Moore, A. M.; Oh, W. C.; Ouyang, Y.; Patel, R.; Paul, M.; Paulsen, L. C.; Qiu, J.; Rhee, A.; Rubashkin, M. G.; Soh, I. Y.; Sotuyo, N. E.; Srinivas, V.; Suarez, A.; Wong, A.; Wong, R.; Xie, W. R.; Xu, Y.; Yu, A. T.; Koszul, R.; Bader, J. S.; Boeke, J. D.; Chandrasegaran, S., Total synthesis of a functional designer eukaryotic chromosome. *Science* **2014,** *344* (6179), 55-8.
4. Messerschmidt, S. J.; Schindler, D.; Zumkeller, C. M.; Kemter, F. S.; Schallopp, N.; Waldminghaus, T., Optimization and characterization of the synthetic secondary chromosome synVicII in *Escherichia coli*. *Front Bioeng Biotechnol* **2016,** *4*, 96.
5. Hochrein, L.; Mitchell, L. A.; Schulz, K.; Messerschmidt, K.; Mueller-Roeber, B., L-SCRaMbLE as a tool for light-controlled Cre-mediated recombination in yeast. *Nat Commun* **2018,** *9* (1), 1931.
